# Collective genomic segments with differential pleiotropic patterns between cognitive dimensions and psychopathology

**DOI:** 10.1038/s41467-022-34418-y

**Published:** 2022-11-11

**Authors:** Max Lam, Chia-Yen Chen, W. David Hill, Charley Xia, Ruoyu Tian, Daniel F. Levey, Joel Gelernter, Murray B. Stein, Alexander S. Hatoum, Hailiang Huang, Anil K. Malhotra, Heiko Runz, Tian Ge, Todd Lencz

**Affiliations:** 1 Division of Psychiatry Research, The Zucker Hillside Hospital, Northwell, Glen Oaks, NY USA; 2 Institute of Behavioral Science, Feinstein Institutes for Medical Research, Manhasset, NY USA; 3 Stanley Center for Psychiatric Research, Broad Institute of MIT and Harvard, Cambridge, MA USA; 4 Analytical and Translational Genetics Unit, Massachusetts General Hospital, Boston, MA USA; 5 Institute of Mental Health, Singapore, Singapore; 6 Translational Biology, Research and Development, Biogen Inc, Cambridge, MA USA; 7 Lothian Birth Cohorts group, Department of Psychology, University of Edinburgh, Edinburgh, UK; 8 Computational Biology and Human Genetics, Dewpoint Therapeutics, Boston, MA USA; 9 Department of Psychiatry, Yale University School of Medicine, New Haven, CT USA; 10 VA Connecticut Healthcare System, West Haven, CT USA; 11 Department of Genetics, Yale University School of Medicine, New Haven, CT USA; 12 Department of Neuroscience, Yale University School of Medicine, New Haven, CT USA; 13 VA San Diego Healthcare System, San Diego, CA USA; 14 Department of Psychiatry, University of California, San Diego, La Jolla, CA USA; 15 Herbert Wertheim School of Public Health and Human Longevity Science, University of California San Diego, La Jolla, CA USA; 16 Department of Psychiatry, Washington University in St. Louis Medical School, St. Louis, MO, USA; 17 Department of Psychiatry, Zucker School of Medicine at Hofstra/Norwell, Hempstead, NY USA; 18 Department of Molecular Medicine, Zucker School of Medicine at Hofstra/Norwell, Hempstead, NY USA; 19 Psychiatric and Neurodevelopmental Genetics Unit, Center for Genomic Medicine, Massachusetts General Hospital, Boston, MA USA; 20 Department of Psychiatry, Massachusetts General Hospital, Harvard Medical School, Boston, MA USA

**Keywords:** Genetic association study, Cognitive neuroscience

## Abstract

Cognitive deficits are known to be related to most forms of psychopathology. Here, we perform local genetic correlation analysis as a means of identifying independent segments of the genome that show biologically interpretable pleiotropic associations between cognitive dimensions and psychopathology. We identify collective segments of the genome, which we call “meta-loci”, showing differential pleiotropic patterns for psychopathology relative to either cognitive task performance (CTP) or performance on a non-cognitive factor (NCF) derived from educational attainment. We observe that neurodevelopmental gene sets expressed during the prenatal-early childhood period predominate in CTP-relevant meta-loci, while post-natal gene sets are more involved in NCF-relevant meta-loci. Further, we demonstrate that neurodevelopmental gene sets are dissociable across CTP meta-loci with respect to their spatial distribution across the brain. Additionally, we find that GABA-ergic, cholinergic, and glutamatergic genes drive pleiotropic relationships within dissociable meta-loci.

## Abstract (web summary)

Cognitive impairments are a key feature of psychopathology. Here, authors exploit the genetic overlap between cognitive dimensions and psychopathology to parse the biology of psychiatric illness and identify “meta-loci” genome segments characterized by specific patterns of overlap.

## Introduction

Cognitive impairment is one of the core features of psychopathology and is associated with the debilitating nature of many psychiatric disorders^1,2^. In schizophrenia, for example, cognitive impairments are predictive of known functional impairments even in the prodromal stage of the illness^3,4^. Cognitive deficits are not only confined to adult psychiatric illnesses, but also extend to childhood disorders like autism spectrum disorders (ASD) and attention-deficit hyperactivity disorder (ADHD)^5^. Individuals who suffer from psychiatric disorders tend to report sequelae of cognitive problems throughout their lifetime^6^. In many cases, there is an emergence of cognitive deficits before a formal diagnosis of mental illness^7^.

Prior to the era of well-powered GWAS in psychiatry, the idea of using cognitive function as an endophenotype to understand the biology of psychopathology was proposed^8^. We then presented the molecular genetic evidence for overlap between cognitive task performance and schizophrenia^9^. Since then, evidence suggesting widespread pleiotropy across psychopathologic traits has emerged, indicating shared biological mechanisms^10–12^. Pleiotropy, a phenomenon where a genetic variant might affect several traits at once, appears to be ubiquitous in biology; 44% of the loci reported within the GWAS catalog have been shown to be associated with more than one trait^13^ (although in some cases this may be a function of vertical pleiotropy or linkage disequilibrium rather than horizontal pleiotropy^14^). A more recent study indicated that trait associated loci cover more than half of the genome, among which 90% implicate multiple traits^15^.

We recently exploited pleiotropy to dissect biology underlying the counter-intuitive positive genetic correlation between educational attainment and schizophrenia, and were able to parse separate neurodevelopmental and synaptic mechanisms underlying the disorder, based on association patterns within GWAS significant loci^16^. Our results complemented earlier findings that (at least) two distinct biological processes appeared to subserve schizophrenia^17^. Shortly after, Demange and colleagues^18^ demonstrated that it was possible to leverage global genetic correlation within a structural equation modeling framework to derive a latent non-cognitive factor (NCF) by removing variance related to cognitive task performance (CTP) from educational attainment GWAS. The ensuing NCF factor showed positive genetic correlation with schizophrenia, consistent with our earlier findings^16^ (It should be noted that the content of the latent NCF construct has not yet been fully specified and may capture, in part, cognitive phenotypes that are separate from CTP, such as reaction time, as well as personality traits such as conscientiousness).

These two reports, utilizing somewhat orthogonal but complementary approaches, leveraged pleiotropic phenomena to study the intersection of cognition and psychopathology. Nevertheless, these studies are limited by following a global genetic correlation approach^19^ on the one hand, and a SNP-by-SNP approach^16,18^ on the other. As has been demonstrated^20^, the assumption that genetic correlations for complex traits are homogeneously distributed across independent genomic regions may not be true. At the same time, typical locus-based GWAS comparisons tend to be defined by ”top” SNPs followed by LD clumping; this invariably results in loss of information from regions of the genome that fall short of genome-wide significance. Studies using partitioned heritability or gene set analysis have demonstrated the biological relevance of regions of the genome that need not contain genome-wide significant loci^21^.

In the present study, we develop a method, intermediate to global and SNP-based approaches, that examines the structure of local genetic correlations across the genome and identifies “meta-loci”, which we define as combined genomic segments sharing pleiotropic patterns. We then interrogate these meta-loci, using rich annotations and gene set analysis, to identify and dissociate biological pathways underlying different patterns of cognitive-psychopathologic pleiotropy.

## Results

### Study design and methods overview

**Fig. 1:**
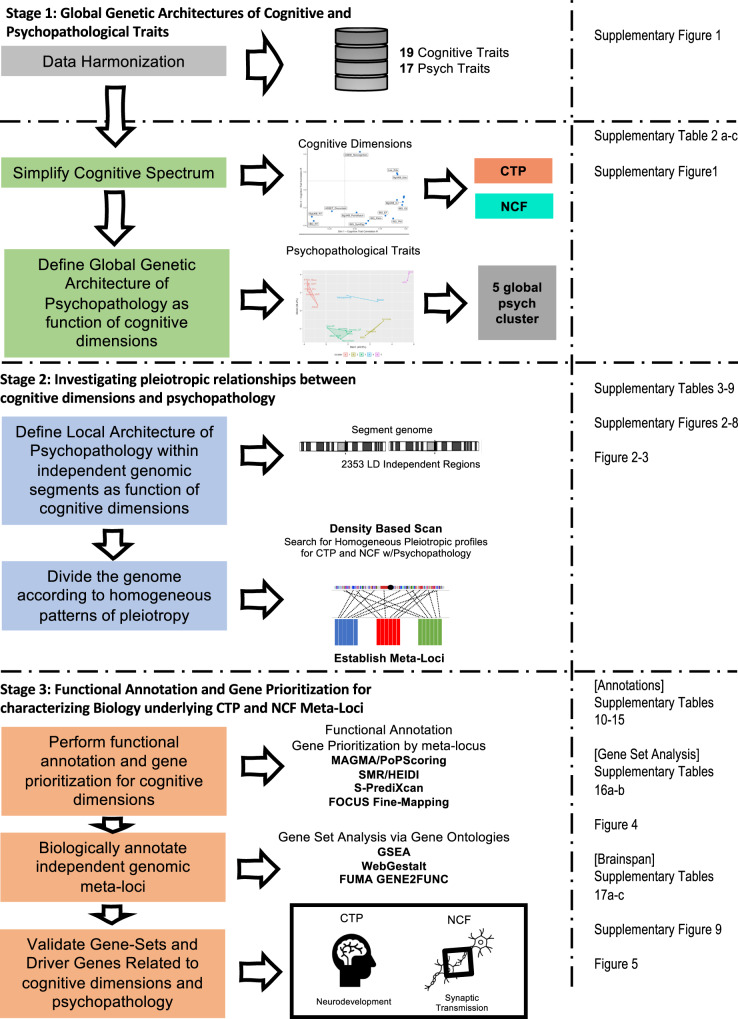
Data analysis workflow. CTP cognitive task performance, NCF non-cognitive factor. The flow chart is divided in three columns. The first column from the left describes the analytic objectives across study stages. The middle column lists the method/software used for the analysis. The right column points the reader to the Table, Figure, Supplementary Data or Supplementary Figure that reports the results of the analysis.

We have recently reported the largest GWAS meta-analysis for CTP (*N* = 373,617) and utilized this well-powered phenotype for pleiotropic analysis with an expanded list of psychopathology phenotypes^22^. Expanding on earlier analytic strategies^16,18^, we carried out three broad stages of analyses (Fig. 1).

First, as both a data reduction step and a benchmark for subsequent steps, we examined the global pleiotropic relationships between multiple cognitive and psychopathological phenotypes via LD score regression^23,24^. We amalgamated all available summary statistics from recent GWAS of cognitive function (*N* = 19). These included CTP^22^, and other reported derivatives of cognition including NCF^18^, Executive Function^25^, and GWAS of individual cognitive tests administered as part of the UK Biobank. Similarly, traits related to psychiatric illnesses or psychopathology in the recent studies from the Psychiatric Genomics Consortium, UK Biobank, Million Veterans Program and elsewhere were curated (*N* = 17; Supplementary Data 1). Data reduction approaches indicated that it was appropriate to focus on CTP and NCF traits for the purpose of the current study (Fig. S1). Specifically, PC1 and PC2 capture the majority of the cognitive GWAS variance (out of 19 cognitive GWAS considered); moreover, CTP and NCF are the strongest individual correlates of PC1 and PC2, respectively (Fig. S1b, c). We re-estimated NCF using the largest CTP GWAS^22^ to increase statistical power (Methods).

Second, we carried out local genetic correlation analyses between each of the psychopathology traits and CTP, and separately between each psychopathology trait and NCF. A series of positive and negative local genetic correlation patterns emerged across these analyses, which we then classified via the “meta-locus” approach. A meta-locus was defined as a set of LD-independent regions that showed similar local genetic correlation profiles across psychopathological traits. There were 15 distinct meta-loci (median length = 123.75 Mb) identified for CTP and 10 identified for NCF (median length = 161 Mb).

Third, CTP and NCF GWAS summary statistics were functionally prioritized to identify genes and biological mechanisms harbored within the meta-loci. A series of gene-based genome-wide association (GBGWA) and transcriptome-wide association (TWA) strategies were applied to CTP and NCF GWAS summary statistics. We leveraged brain eQTLs from a range of databases, including GTEx v8 brain tissue expression^26^, Brain-eQTL-meta^27^, and PsychEncode^28,29^ eQTL databases that index brain (Online Methodology and Supplementary Information). We adopted a broadly inclusive approach to the gene prioritization stage of the analysis, excluding from consideration only those genes with no supporting evidence from any of these procedures. Next, we performed a series of gene set analyses^30–33^ and gene scoring procedures^34^, to which we applied strict filtering criteria in order to arrive at a high-confidence biological characterization of each meta-locus. We evaluated the high-confidence genes emanating from the GBGWA, TWA and gene set analysis approaches for longitudinal gene expression across lifespan to further differentiate neurodevelopmental vs adult functional mechanisms, and further examined regional distribution of these genes across the brain. Finally, we annotated “driver genes” (the core of a gene set accounting for the enrichment signal) with information on the propensity for psychiatric or nootropic drug re-purposing (Fig. 1 and Methods).

### Stage 1: Global genetic architectures

As an initial data reduction step, due to the very high level of overlap between many of the cognitive measures, a wrapper function within GenomicSEM^11^ was used to conduct LD score regression^23,24^ and create a global 19 × 17 genetic correlation matrix of cognitive and psychopathological traits (Supplementary Data 2). Two dimensionality reduction techniques, PCA and partitioned cluster analyses (K-Medoid), were applied to the global genetic correlation matrix to identify underlying pleiotropic patterns. PCA and partitioned cluster analysis indicated that CTP was most pleiotropic with psychopathological traits followed by NCF (Fig. S1a). The first principal-component captured the similarity of each cognitive trait to cognitive task performance (CTP) in context of its relationship to the vector of 19 psychopathological traits (Fig. S1b). The second principal-component represented the degree to which a cognitive trait is similar to NCF given its relationship to the vector of 19 psychopathological traits (Fig. S1c). Consequently, for all subsequent analyses, we focused on CTP and NCF as the primary cognitive phenotypes of interest; we leave a more detailed exploration of the cognitive phenotypic space to future work. We re-extracted the NCF latent factor by using GWAS-by-subtraction parameters^18^, and a better-powered CTP meta-analysis^22^. We confirmed that our newly calculated CTP and NCF factors were globally similar to those originally reported by Demange et al.^18^ (*r*_g_ = 1 using LD score regression). We utilized the current versions of CTP and NCF in subsequent sections detailing functional annotation, gene prioritization and gene set analyses. At the global level, we also observed that psychopathological traits separated into five best-fitting clusters (Supplementary Data 2c) that varied according to the degree of the strength of relationship with CTP and NCF (Fig. S1d, inset). There was much more variation across psychopathological traits than the cognitive traits, so we decided to analyze each trait separately in the local genetic correlation analyses described below. We utilized the clustering results depicted in Fig. S1d as background information for our interpretations of subsequent results (and as color-coding in subsequent figures in this report).

### Stage 2: Local genetic correlations to investigate pleiotropic relationships

Local genetic correlation analyses were carried out across 2353 LD-independent regions of the genome using ρ-HESS^20^. 4,469,149 SNPs were included for CTP and 4,372,398 SNPs were included for NCF. Region-specific heritability estimates of CTP and NCF were expectedly small (median *h*^*2*^_CTP-Region_ = 8.36e-5, median *h*^*2*^_NCF-Region_ = 1.18e-4, Supplementary Data 3). The sum of heritability across LD independent regions was consistent with previous reports for these phenotypes (*h*^*2*^_CTP_ = 0.23, *h*^*2*^_NCF_ = 0.31) (See Methods), except in the case of anorexia nervosa and OCD, for which ρ-HESS recovered a greater degree of correlation with NCF (Fig. S2).

Local genetic correlations showed widespread pleiotropy for both CTP and NCF with psychopathology (Supplementary Data 4 & Supplementary Data 5). Aside from re-capitulating global *R*_g_ trends across psychopathology phenotypes, several noteworthy observations emerge from the local genetic correlations. First, for each psychopathological trait, local genetic correlations were not always in the expected direction, across LD-independent regions, compared to global genetic correlations (Fig. S3a, b). Second, the magnitude of the local genetic correlation signals is markedly stronger for schizophrenia than for any other trait. Third, schizophrenia is the only trait (with the potential exception of anxiety, see “Anxiety MVP”) that demonstrates strong, widespread differences in direction between CTP and NCF correlations (Fig. S3 and Supplementary Data 6). Fourth, local genetic correlations for CTP were especially strong within the Major Histocompatibility Complex (MHC); notably, anorexia nervosa and ADHD show the opposite pattern of local genetic correlations within the MHC locus relative to other psychopathological traits (see Fig. S4).

To examine pleiotropy across multiple traits and regions in the genome, we first started with the local genetic correlation matrices (17 psychopathologic traits x 2330 LD-independent regions, excluding the MHC) for CTP and NCF, respectively (Supplementary Data 4 and Supplementary Data 5; these are two separate 2353 × 17 matrices). We then reduce the high-dimensional data using Uniform Manifold Approximation and Projection for Dimension Reduction (UMAP) to derive 20-dimensional features for CTP and 10-dimensional features for NCF; the number of features extracted was determined empirically for each of the two phenotypes based on optimized fit statistics. Clusters within the resulting UMAP space were then identified using a two step-process: Density-Based scanning (DBSCAN) sequential clustering (“bottom-up” approach) yielded intermediate localized clusters (bottom-up clustering) for CTP and NCF; this initial clustering solution was then optimized with more commonly used hierarchical clustering methodology (“top-down approach”).

**Fig. 2:**
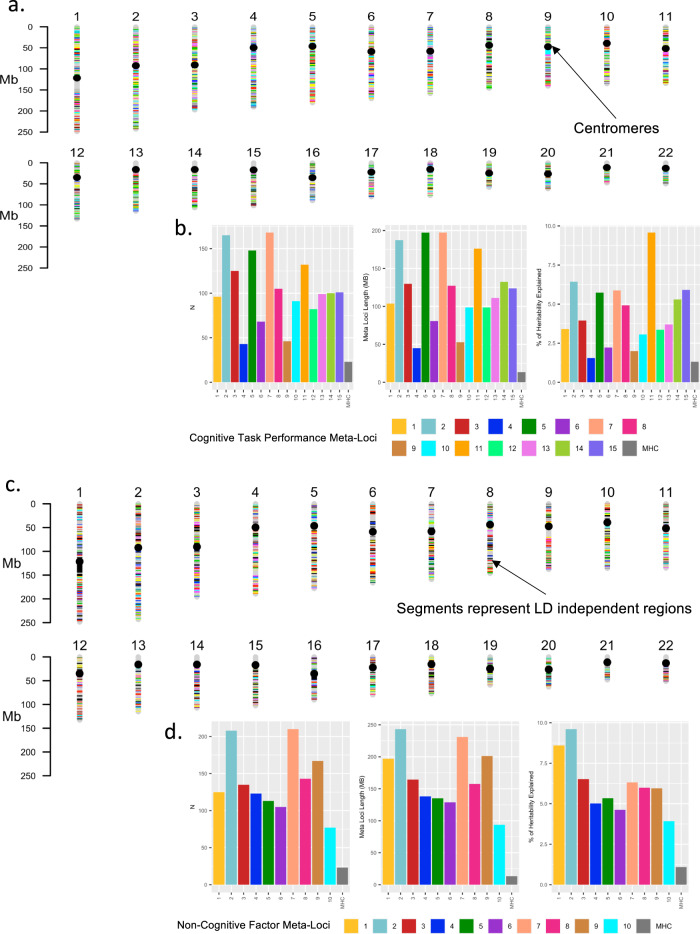
Karyotype plots for genomic locations of meta-loci and descriptive statistics for meta-loci. **a** Karyotype plot for CTP (**b**) number of LD segments, length, and percentage of heritability estimated for each meta-loci for CTP (**c**) karyotype plot for NCF (**d**) number of LD segments, length and percentage of heritability estimated for each meta-loci for NCF.

The resulting final set of clustered genomic regions, each of which contained between 43 to 210 LD-independent segments (ranging in total length from 45MB to 243 MB) are termed “meta-loci”; these meta-loci represent clusters of LD-independent regions with distinct pleiotropic patterns for CTP and NCF, respectively, across the various psychopathologic traits. Importantly, both UMAP and DBSCAN methodologies were calibrated before we applied them to the local genetic correlation matrices, and the meta-loci demonstrated good-excellent stability, uniformity and separation (see Methods for further discussion on investigating latent structure, and details for meta-loci identification; see also Supplementary Data 8, Supplementary Data 9, and Fig. S5). We identified 15 meta-loci for CTP and 10 meta-loci for NCF (Fig. 2; Methods). Critically, the extracted meta-loci were not simply a function of highly localized effects but were distributed across the genome (Fig. 2). Characteristics of each of the meta-loci shown in Fig. 2 demonstrate that that LD independent segments are generally similarly distributed between CTP and NCF phenotypes.

**Fig. 3:**
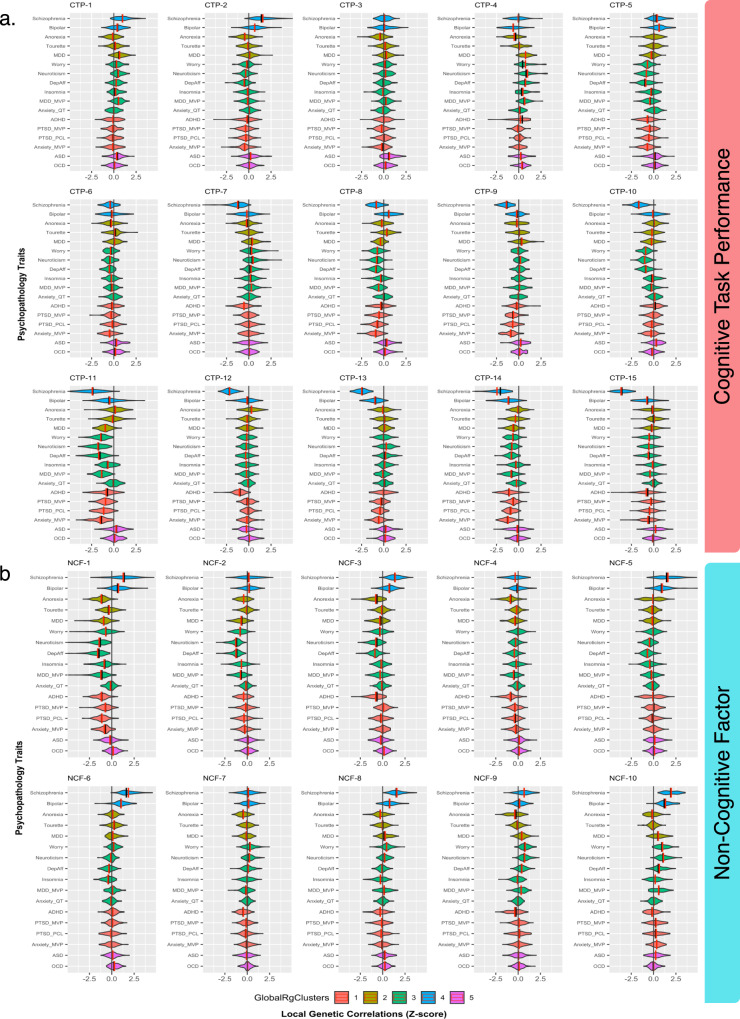
Violin plots for *Z*-score distributions of local genetic correlations within each meta-locus. GlobalRgClusters: Phenotype clusters derived from partitioned clustering of global genetic correlation. CTF cognitive task performance, NCF non-cognitive factor. **a** Prioritized meta-loci for cognitive task performance. **b** Prioritized meta-loci for non-cognitive skills. **a**, **b** Bipolar: bipolar disorder, Anorexia: anorexia nervosa, Tourette’s: Tourette’s syndrome, MDD: major depressive disorder (Howard et al., 2019)^63^, Dep-Aff: depressive-affect, MDD_MVP: major depressive disorder (Million Veteran Project), Anxiety_mvp: anxiety disorder (Million Veteran Project), PTSD_mvp/pcl: post-traumatic stress disorder (Million Veteran Project; Total PCL: total PCL symptom scores).

The distribution of local genetic correlations for LD segments included in each meta-locus between the psychopathological traits and CTP or NCF, respectively, is displayed in Fig. 3 as violin plots; in addition, clustered Manhattan plots for local genetic correlation by meta-loci are also displayed in Figs. S6 and  S7 for CTP and NCF, respectively. As expected, schizophrenia showed strong trends of negative genetic correlation in most CTP meta-loci; it is notable, however, that positive local genetic correlations were observed for 3 of the 15 CTP meta-loci. Schizophrenia and bipolar disorder showed similar local genetic correlation profiles for the NCF meta-loci but were differentiated on several CTP meta-loci. Attention-deficit/hyperactivity disorder (ADHD) was negatively associated with CTP and NCF across meta-loci, whereas obsessive compulsive disorder (OCD) was positively associated with both cognitive dimensions across all meta-loci, except for a few showing no association (*z*~0). Affective and anxiety traits showed expected broad negative associations with both cognitive dimensions. Autism spectrum disorder (ASD) demonstrated a relatively unique pattern of relationships, with relatively modest effects across most meta-loci, except for a positive genetic correlation with CTP at meta-locus CTP-1.

It was important to determine if the pattern of results was driven by socioeconomic status, given that prior literature^35^ has indicated that educational attainment exhibits shared biology with socioeconomic status. ρ-HESS analyses were carried out between CTP and NCF with the Townsend Deprivation Index to examine the association with socioeconomic status (SES); preliminary results regressing CTP and NCF *Z*-scores indicate that neither CTP or NCF is systematically associated with SES (*R*^2^ = 0.0039) (See Supplementary Data 6). As displayed in Fig. S8, most meta-loci (except NCF-1) showed only modest associations with socioeconomic status; Supplementary Data S6 further shows that few chromosomal regions demonstrated strong (*Z* ≤ −2) local genetic correlations with the Townsend Social Deprivation index, and these were not concentrated in any single meta-locus (except again for NCF-1). Thus, confounding with socioeconomic status appears insufficient to account for the relationships observed for individual meta-loci with psychopathologic traits displayed in Fig. 3.

### Stage 3: Functional annotation and gene prioritization for CTP and NCF-meta-loci

To characterize each meta-locus, we applied a series of gene-based and TWAS methods to the CTP and NCF GWAS summary statistics (MAGMA and PoPs gene-based results: Supplementary Data 10; SMR, S-PrediXcan and FOCUS fine-mapping TWAS results: Supplementary Data 11, Supplementary Data 12, & Supplementary Data 13). As an initial loose filter, genes were ranked based on multiple complementary transcriptome analyses (Supplementary Data 14), applying a 50th percentile cutoff for gene association p-values; any genes with no evidence of transcriptomic association to the cognitive phenotypes were removed from further consideration. PoPs gene prioritization scores^34^ was inverse-ranked (Supplementary Data 15) for each remaining gene within a given meta-locus, and then subjected to gene set analyses, using annotations obtained from the GO Gene Sets (Cellular Component, Molecular Function and Biological Process) included in the Molecular Signature Database v 7.2^36^. Detailed descriptions of the parameters applied to each of the methods are described in the Methods section. Gene-set analyses were carried out via three methods [Gene-Set Enrichment Analysis (GSEA^31^), WebGestalt^32^, and GENE2FUNC (part of the Functional Mapping and Annotation of Genetic Association—FUMA—pipeline^33^)].

**Fig. 4:**
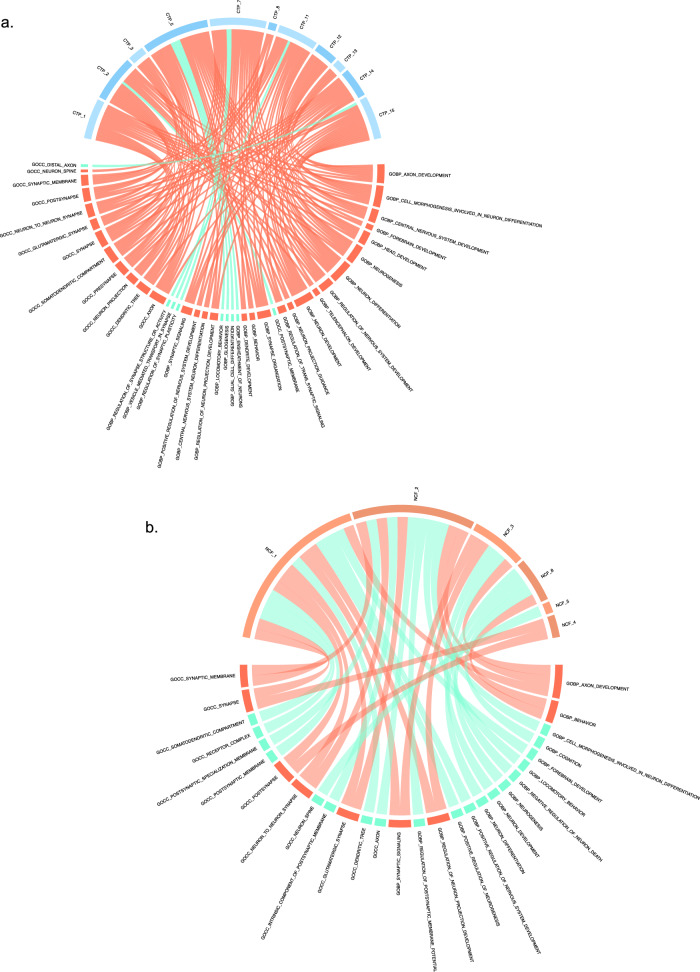
Unique and overlapping gene sets within cognitive task performance and non-cognitive factor meta-loci. CTP cognitive task performance. NCF non-cognitive factor. **a** Circle plot for general cognitive ability and notable meta-loci. **b** Circle plot for non-cognitive skills and notable meta-loci. Turquoise lines within each circle plot represents unique gene sets to either general cognitive ability or non-cognitive skills. Orange lines within the circle plot indicates that the gene set overlaps across both cognitive task performance and non-cognitive factor.

Focusing on gene sets specific to CTP vs NCF meta-loci, or those specific to individual meta-loci, might allow more targeted parsing of the biological mechanisms underlying the relationship between psychopathology and cognition. While numerous brain-related gene sets were observed across meta-loci (Fig. 4 and Supplementary Data 16), several notable distinctions were observed as well. First, there were multiple gene sets featuring a predominately neurodevelopmental theme that were shared across multiple CTP meta-loci yet not observed in NCF meta-loci; these included “GOBP Central Nervous System Development,” “GOBP Head Development,” and “GOCC Neuron Projection.” By contrast, gene sets related to actin filament-based processes (essential for synaptic structure and function) and apoptotic signaling were associated with multiple NCF meta-loci, but no CTP meta-loci. Moreover, we observed a dissociation between CTP and NCF meta-loci with respect to synaptic gene sets; the GO cellular component gene set localized to the presynaptic component was associated exclusively with several CTP meta-loci (CTP-1, CTP-5, CTP-7, CTP-11, and CTP-14), whereas several postsynaptic gene sets were exclusive to one specific NCF meta-locus, NCF-2.

**Fig. 5:**
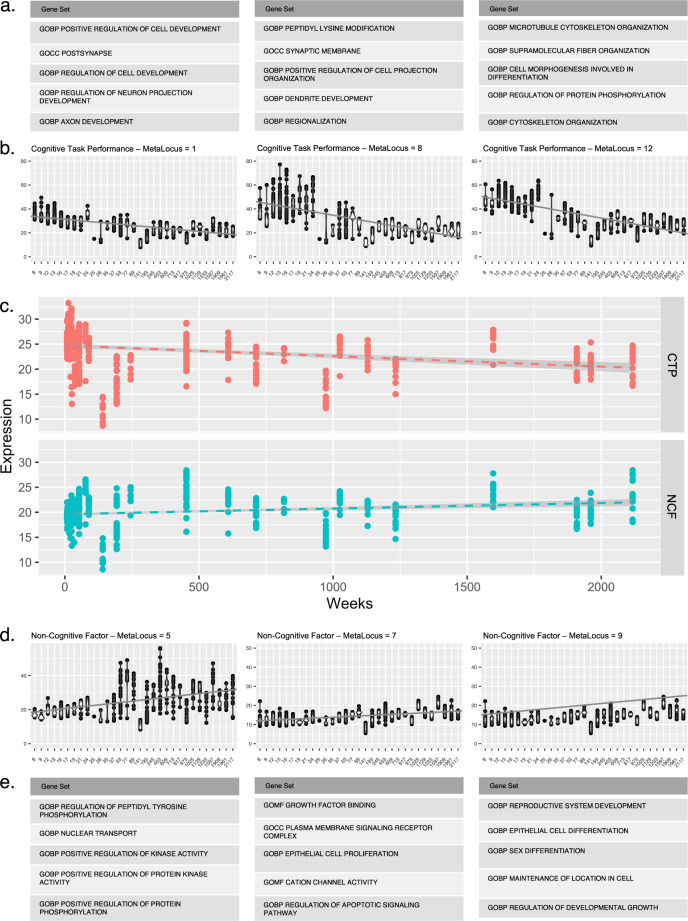
Temporal gene expression within prenatal cognitive task performance and adulthood non-cognitive factor meta-loci. CTP cognitive task performance. NCF non-cognitive factor. **a** Top cognitive task performance gene sets for each indicated meta-locus **b** meta-loci showing significant trajectories for prenatal gene expression implicating cognitive task performance **c** aggregated temporal gene expression for cognitive task performance and non-cognitive factor across meta-loci **d** meta-loci showing significant trajectories for adulthood gene expression implicating non-cognitive factor **e** top non-cognitive factor gene sets for each indicated meta-locus.

The dichotomy between neurodevelopmental and apoptotic pathways underlying CTP and NCF, respectively, suggested an additional hypothesis to be tested. If CTP was primarily driven by neurodevelopmental genes, we would expect to see gene expression profiles that are active prenatally or early in the lifespan, whereas for NCF, genes responsible for synaptic structure and function as well as apoptosis are likely to be expressed later in post-natal life. Leveraging the BrainSpan dataset^37^, which characterized brain expression profiles at various developmental stages, we tested if CTP and NCF driver genes might demonstrate differential expression across the lifespan. By fitting a linear mixed model, with individual as random effect, cognitive phenotypes and time as fixed effects and sex as a covariate, we found developmental differences between CTP and NCF driver genes (for further details, see Methods, results for BrainSpan analysis: Fig. 5 and Fig. S9). Setting the null model simply with time as predictor, we observed a significant improvement in model fit when an indicator of the cognitive phenotype (CTP vs. NCF) for the driver gene was included in the model (*χ*^2^ = 595.74, df = 2, p = 4.33 × 10^−130^). A main effect for differences in CTP and NCF genes was found (*β* = −5.07, se = 0.18, *p* = 1.96 × 10^−130^), and an interaction between CTP and NCF genes with time (weeks) (*β* = 0.0032, se = 0.0002, *p* = 2.46 × 10^−^^41^) was also observed (Fig. 5 and Fig. S9). As hypothesized, genes harbored within NCF-meta-loci were expressed predominantly in early adulthood and adulthood, whereas CTP meta-loci genes were expressed prenatally.

As shown in Fig. 4, brain-related gene sets such as “Axon Development” and “Neurogenesis” were significantly enriched across numerous CTP meta-loci. While these biological pathways are commonly enriched in studies of cognitive and neuropsychiatric phenotypes, our meta-locus approach also permits a further dissection of these gene sets, beyond that permitted by conventional genome-wide analysis. Specifically, the individual genes driving the enrichment of a given set necessarily differ across meta-loci, since the meta-loci are mutually exclusive carvings of the genome. We can then examine the spatial distribution of those genes, using data from BrainScope, to determine if a given biological pathway manifests regional differences across the brain (Figs. S10–S14). For example, the gene set “Axon Development” is significantly enriched in 7 CTP meta-loci. As shown in Fig. S10, the genes comprising this enrichment for meta-locus CTP-1 are broadly expressed in cortex, whereas those for meta-locus CTP-5 are concentrated in the parahippocampal gyrus, hippocampus, and subcortical structures; by contrast, genes involved in axon development for meta-locus CTP-2 are distributed across all brain compartments including the white matter. Similar patterns are observed across other neurodevelopmental gene sets (Figs. S11–14).

Finally, we further annotated each meta-locus as a function of individual “driver” genes, unique to each CTP or NCF meta-locus, as identified from the GSEA analysis. These genes were further examined if they were potentially actionable in terms of encoding proteins for putative drug targets, using chemoinformatic annotations provided by Finan et al.^38^ (Supplementary Data 15). For each meta-locus, Tier 1 druggable genes (i.e., genes with current evidence of having existing compounds that are FDA approved and being utilized for various indications), summarized alongside other information specific to each meta-locus, are listed in Tables 1 and  2. For example, multiple glutamatergic genes, as well as *DRD2* (dopamine D2 receptor) were significant drivers at the CTP-11 meta-locus, in which genes reducing cognitive test performance are strongly associated with risk for schizophrenia (as well as many other forms of psychopathology). By contrast, the gene encoding the glycine transporter (*SLC6A9*) is a significant driver at CTP-15, which is almost exclusively associated with schizophrenia risk. Notably, the NCF-2 meta-locus implicates several GABA receptor genes, as well as the GABA transporter *SLC6A1*, suggesting the possibility that GABA-ergic treatment approaches may enhance NCF while simultaneously ameliorating the correlated affective and anxiety symptoms that load on this meta-locus. This interpretation of NCF-2 is further strengthened by results of drug-based gene set analysis conducted using WebGestalt^32^ (final column of Tables 1 and 2; full details in Supplementary Data S16b). Two anti-epileptic medications commonly used for the treatment of bipolar disorder, lamotrigine and valproic acid, are significantly enriched in the drug-based gene set analysis for NCF-2; both medications have indirect/downstream effects on GABA transmission^39^. Similarly, multiple cholinergic genes are implicated at CTP-2, and nicotine was significant in the drug-based gene set analysis for this meta-locus.

**Table 1:** Cognitive task performance (CTP) meta-loci, biological and drug gene sets, and druggable gene targets

Meta-locus ID	*N* LD segments	*h*^2^	Length (MB)	Biological gene sets^a^	Druggable genes^c^	Drug-based gene sets^b^
1	96	0.0079	103.73	GOBP response to growth factor GOBP transmembrane transport GOBP ion transmembrane transport	ABCC5 APH1B ATP1A3 BCL2 DCLK1 DRD4 ESR2 GRIK5 GSK3A HRAS PLK1 PRKCB	-
2	165	0.0149	187.30	GOBP cell cycle GOBP cell cycle process GOCC vesicle membrane	BCL2L1 BMPR1B CDK19 CHRM2 CHRNA3 CHRNA4 CHRNB4 CSNK1E GABRA1 GABRG2 GRIA4 KCNQ2 LAMC3 MAP3K7 MET NPC1 OPRM1 PDE4D ROCK1 RPS6KA5 RTN4 RXRG SOD1 STK25 TPBG TUBB4A	TROGLITAZONE; PHENOL; EZOGABINE; NICOTINE; HESPERETIN; PROPOFOL; 4-METHYLUMBELLIFERONE; MYCOPHENOLATE_MOFETIL; ISOTRETINOIN
3	125	0.0091	129.75	GOBP carbohydrate homeostasis GOMF TRansferase Activity Transferring Phosphorus Containing Groups GOBP response to lipid	ABL2 CAMK2G DNMT1 DPYD EPHA6 HSP90AA1 INSR MAP3K3 MAP3K9 MAPK13 MAPK14 MARK3 NTRK3 PDE4A PDPK1 PKN1 PRKACA PRKCE SIRT1 SRPK1 TESK1 TYK2	ANGIOTENSIN_II_ANTAGONISTS
4	43	0.0036	44.87	-	-	-
5	148	0.0132	197.22	GOBP locomotory behavior GOBP locomotion GOBP animal organ morphogenesis	AKT3 BCR BRAF CAMK2A CD74 CHST11 CLK1 CSF1R CSNK1A1 DRD3 DSTYK DYRK1A ERBB4 GAK GRIN3A HIPK2 HTR1A INHBA L3MBTL1 MAP2K1 MAP2K7 MAPK3 MINK1 MMP17 MTOR PAK2 PDE10A PHKG2 PIP4K2B PPARA PRKAA2 PRKCA PSENEN TSHR ULK1 VKORC1	PROTEIN_KINASE_INHIBITORS DRUGBANK CNS AGENTS
6	68	0.0051	80.64	-	-	CITRIC_ACID ALPHA-LINOLENIC_ACID QUININE
7	168	0.0136	197.24	GOMF RNA binding GOCC golgi membrane GOBP regulation of lipid metabolic process	AKT1 CDK4 CHST8 EIF2AK4 EIF4E EPHB6 FKBP1A FKBP4 MAP3K4 METAP1 MKNK1 PCSK9 RXRA SLC6A1 STK33 TP53	-
8	105	0.0114	127.26	GOBP embryo development	APH1A ARNT CHRNB2 MAP3K13 PIP5K1A SIGMAR1 SV2A	-
9	46	0.0051	80.64	-	-	-
10	91	0.0071	98.62	-	AHR CTSB DNMT3A GRIN2C HDAC9 HMGCR	-
11	132	0.0221	176.15	GOCC presynaptic membrane GOMF ubiquitin like protein transferase activity GOBP regulation of cell death	BACE1 CACNA1D CAMKK2 CCNB1 CDK7 CDK9 CIT CXCR5 DAPK3 DMPK DRD2 EEF2 EGFR EPHA10 EPHB1 GIPR GRIK1 GRIK3 GRM2 HDAC5 HRH3 IMPDH2 ITGA2B KMT2A MAP2K2 MAP2K6 MARK4 NISCH P4HTM PARP1 PDE4C PIK3CA POLD1 PRKD1 PSMB2 PTK2 SLCO3A1 VAMP2	-
12	82	0.0078	98.69	GOBP microtubule-based process	CNR1 CSNK1D DAGLB FASN HDAC2 NCFTN PIK3CB	AMIFAMPRIDINE
13	99	0.0085	111.18	-	EPHA5 EPHA8 EPHB2 HIPK1 MAP4K4 TAOK3 TNIK	-
14	100	0.0122	132.29	GOBP RNA splicing via transesterification reactions GOMF actin binding GOBP supramolecular fiber organization	ABCC8 CACNA1B CACNA1G GRIN1 GRM3 HDAC1 MARK2 NOTCH1 PAK6 POR PPP3CA PRKCI RAF1 STAT3 ULK2	-
15	101	0.0137	123.75	GOBP cellular response to DNA damage stimulus GOBP DNA metabolic process GOBP-negative regulation of protein metabolic process	AKT2 BRSK2 CACNA1I DYRK1B EGLN2 GPX4 HDAC3 MAP3K10 MAP3K12 MARK1 NTRK2 PDE7B PRKCG SLC6A9 SRPK2 STK11 TLK2	-

^a^Top 3 biological gene sets, ranked by lowest nominal marginal *p*-value for each meta-locus.

^b^Drug-based gene sets selected based on DrugBank annotations and FDR  <  0.05 significance levels for gene set analysis, as well as potential for nootropic function. Oncology drugs sets are excluded due to complex drug delivery procedures.

^c^Tier 1 druggability annotations from Finan et al. (2017)^38^.

**Table 2:** Non-cognitive factor (NCF) meta-loci, biological and drug gene sets, and druggable gene targets

Meta-locus ID	*N* LD segments	*h*^2^	Length (MB)	Biological gene sets^a^	Druggable genes^c^	Drug-based gene sets^b^
1	125	0.0271	196.85	GOBP appendage development GOBP cell–cell junction organization GOBP cell fate commitment	PDPK1 AKT3 APH1A ARNT PIP5K1A SV2A IMPDH2 P4HTM EPHA5 ADORA2A ANGPT1 CACNA2D2 ESR1 GABRA2 GABRA4 GABRB1 GABRG1 GPX1 GRIN2B LAMB2 LAMC1 MAPT MST1R VEGFA	CIPROFLOXACIN^d^
2	208	0.0303	243.30	GOBP regulation of postsynaptic membrane potential GOBP intrinsic apoptotic signaling pathway GOBP sensory organ morphogenesis	HRAS GABRA1 GABRG2 PDE4D RXRG CAMK2G EPHA6 PKN1 SLC6A1 TP53 BACE1 CIT KMT2A PTK2 TNIK NTRK2 CACNA1C CHRM1 CHRNB1 CSK DAPK2 DDO GABRA6 HDAC7 HIF1A KIT NCOR2 NT5E PDE7A SNAP25 TNNI3K	VALPROIC_ACID^d^; POLYMYXINS^d^; LAMOTRIGINE; PROPOFOL; PURINE_DERIVATIVES
3	135	0.0205	164.43	GOBP metal ion transport GOBP-positive regulation of intracellular transport GOBP regulation of transmembrane transport	PLK1 PRKCB CHRM2 CACNA2D1 CASP1 CCND1 CDK13 CHRND CHRNG GRIN2D GSK3B HCRTR2 JAK2 MAG NFKB1 SIK3	-
4	123	0.0158	138.01	GOBP-negative regulation of intracellular signal transduction GOBP-positive regulation of intracellular signal transduction GOBP response to extracellular stimulus	CHRNA3 CHRNB4 OPRM1 ABL2 NTRK3 CDK4 RXRA PRKD1 VAMP2 SLC6A9 AURKB BCHE CYP27B1 EPHA1 GPBAR1 GPR17 IL1A KCNN4 MAST1 MGMT OPRD1 PDE4B S1PR4 TNK2	ANAKINRA
5	113	0.0168	135.20	GOBP regulation of peptidyl tyrosine phosphorylation	RPS6KA5 PRKCA HDAC2 HDAC1 MARK2 PPP3CA CHRNA2 DRD1 ESRRA GRM5 ITGB3 LMNA MAP3K11 MAP4K2 MUC1 PAK1 PDE5A PSEN1 PTK2B SCN4A VEGFB	SILVER^d^
6	105	0.0146	128.79	-	-	-
7	210	0.0199	231.07	GOBP glucose metabolic process GOMF cation channel activity	CHRNB2 CACNA1I CHRNE ERBB2 GRIA2 HTR1B ITGAV NGFR	L-PROLINE; TOLBUTAMIDE; SARILUMAB
8	143	0.0189	157.57	GOBP tube development GOCC cell surface GOBP striated muscle cell differentiation	ESR2 CSNK1E NPC1 ROCK1 DNMT1 PDE4A PRKCE TESK1 DRD3 GRIN3A HDAC5 ITGA2B ACVR1 DPP9 EPHA2 FSHR GABRB2 ICAM1 ICAM3 LHCGR PDE6C ROCK2 SOD2 TNKS	HEROIN^d^; L-ALANINE
9	167	0.0187	201.30	GOBP sex differentiation	SOD1	CARBONIC ANHYDRASES; TRIHEXYPHENIDYL^d^; DEUTETRABENAZINE; MYRRH; PHENELZINE
10	77	0.0124	93.58	-	-	-

^a^Top 3 biological gene sets, ranked by lowest nominal marginal *p*-value for each meta-locus.

^b^Drug-based gene sets selected based on DrugBank annotations and FDR  <  0.05 significance levels for gene set analysis, as well as potential for nootropic function. Oncology drugs sets are excluded due to complex drug delivery procedures.

^c^Tier 1 druggability annotations from Finan et al. (2017)^38^.

^d^Drug reported to be associated with cognitive impairment rather than nootropic function.

## Discussion

In the last several years, the accumulation of evidence from single-trait GWAS has pointed broadly toward neurodevelopmental and synaptic biology for psychiatric phenotypes, although gene set analyses of individual psychiatric disorders have sometimes failed to identify one^40,41^ or both^42^ of these pathways. Other single-trait GWAS in psychiatry have implicated both these broad mechanisms, but without the ability to dissociate more refined biological subsets^43,44^. Similarly, while numerous recent studies have demonstrated shared genetic underpinnings amongst multiple forms of psychopathology, as well as between psychiatric and cognitive phenotypes^10,11,25,45^, these studies have provided limited additional biological insights concerning the sources of this pleiotropy^10,11,15^ (Supplementary Data 18). As cognitive impairment is a nearly ubiquitous feature of psychopathology, and cognitive performance is a robust clinical indicator of brain function, we hypothesized that leveraging cognitive phenotypes along with pleiotropic investigation of psychopathological traits was an approach that could aid in dissecting the neuropsychiatric biology. The present study was designed to parse the genetic overlap into separable biological pathways with specific psychiatric and cognitive subcomponents. The present study extends our previous work, in which we leveraged cognitive pleiotropy to differentiate early neurodevelopmental mechanisms from adult synaptic dysfunction in the etiopathogenesis of schizophrenia^16^.

Here, we demonstrated that prenatal neurodevelopmental mechanisms shape the relationship between cognitive task performance and multiple forms of psychopathology, while pathways expressed later in life underlie the paradoxical association between higher scores on a non-cognitive factor (relating to educational attainment) and greater risk for psychotic disorders. Moreover, we were able to further specify individual gene sets with distinct patterns of association to psychiatric and cognitive/non-cognitive phenotypes, and we localized some of these gene sets to specific brain regions. As an example, we demonstrated specific involvement of genes expressed subcortically in CTP-5, which was primarily marked by loadings for affective and anxiety-related traits, whereas genes that were expressed in a more widespread distribution were associated with meta-loci featuring strong loadings for psychotic disorders.

The discoveries in the current report were made possible by our construction of “meta-loci”, defined by the concatenation of LD-independent genomic regions with shared patterns of local genetic correlations across phenotypes. Within meta-loci, we further identified genes that may serve as actionable targets, or at least actionable entry points into relevant biological pathways, for psychiatric and nootropic drug re-purposing (Tables 1 and 2); a few examples are described below.

The CTP-11 meta-locus was characterized most significantly by the “Presynaptic membrane” gene set, with notable driver genes including *DRD2*. While the *DRD2* locus is significant in GWAS of schizophrenia^46^, and the dopamine D2 receptor plays a central role in antipsychotic medications^47^, the pattern of local genetic correlations at CTP-11 suggest that this mechanism is pleiotropic across multiple forms of psychopathology, consistent with the utility of D2-antagonizing antipsychotic medication in the treatment of bipolar and unipolar affective disorders. Similarly, we found that CTP-8, characterized most strongly by “Dendrite Development” and “Embryonic Development” gene sets, was associated with most forms of psychopathology. Driver genes in this meta-locus included *DCC* and *CTNNA2*, among many known genes that implicate psychopathology. In their most recent report, the PGC Cross Disorder group reported that the *DCC* region was most pleiotropic in psychopathology. The *DCC* gene was a known master regulator of early neurodevelopmental biology via interactions with netrin-1 and draxin and has been implicated in developmental processes of white matter tracts in the brain^48,49^. Consequently, the PGC authors suggested that *DCC* is likely to affect childhood developmental disorders such as ADHD and ASD, which they have demonstrated to cluster together. Importantly, our evidence showed that similar processes are not limited to neurodevelopmental disorders but are implicated in other psychopathological phenotypes and are linked through a shared effect on cognitive task performance.

By contrast, meta-locus CTP-3, which spans 130MB across 165 segments, demonstrated a unique pattern of pleiotropy, in which cognitive task performance was positively associated with risk for autism, yet inversely associated with risk for anorexia. As shown in Table 1 (and in more detail in Supplementary Data 16), this meta-locus is uniquely characterized by genes associated with carbohydrate metabolism and response to lipid, including the insulin receptor gene *INSR*. This finding is consistent with recent GWAS evidence indicating that anorexia is (in significant part) a metabolic disorder^50^, and extends this finding to demonstrate pleiotropy of this result with cognitive performance and autism. Similarly, an association between risk for anorexia and lower non-cognitive factor scores at meta-locus NCF-7 is driven (in part) by the gene set representing glucose metabolic processes.

The meta-locus approach also extends and refines prior work on the structure of psychopathology. Recently, both the PGC Cross Disorder Group^10^, as well as Grotzinger and colleagues^45^ utilized global genetic correlations to show converging evidence for several latent factors underlying current nosological constructs in psychiatry: (i) Psychosis factor (schizophrenia and bipolar disorder); (ii) Neurodevelopmental factor (alcohol use, ADHD, ASD, PTSD); (iii) Compulsive factor (anorexia nervosa, OCD, and Tourette’s Syndrome); and (iv) Internalizing factor (MDD, anxiety disorders). In the current report, schizophrenia and bipolar disorder generally demonstrated congruent patterns of pleiotropy globally, consistent with the factor structure described above; nevertheless, distinctions were observed at several meta-loci (e.g., CTP-8), where schizophrenia more closely resembled major depression and other affective phenotypes. Further, schizophrenia appears to harbor LD-independent segments strongly associated with CTP beyond that of other psychopathological phenotypes. These results support the notion that schizophrenia is potentially a more cognitively loaded disorder compared to other psychiatric conditions, including bipolar disorder. Though the genetic correlation between schizophrenia and cognitive dimensions appears modest (*r*_g_ ≈ −0.2) at the global level, this may reflect mutually negating effects at the regional level across the genome, as indicated by counter-intuitive-positive correlations between cognitive task performance and risk for schizophrenia noted at CTP-1 and CTP-2. Broad pleiotropic profiles for CTP/NCF within ADHD, PTSD, MDD, Anxiety disorder and Tourette’s Syndrome support cross factor loadings observed by Grotzinger and colleagues^45^ between “Internalizing” and “Neurodevelopmental factors”. However, in the present study, ADHD and ASD exhibited quite different local genetic correlation profiles across most meta-loci—ADHD being negatively correlated across nearly all meta-loci for CTP and NCF, while ASD demonstrated null or positive correlations with both CTP and NCF meta-loci.

Although the MHC region was excluded from most of the downstream work due to its complicated LD patterns (and resulting challenges in distinguishing relevant genes and gene sets), it is worth mentioning that the MHC showed stronger CTP-psychopathology local genetic correlations than other genomic regions. Also noteworthy is that anorexia nervosa and Tourette’s syndrome showed opposite local genetic correlations within the MHC region relative to all other CTP/NCF—psychopathology trait pairs. Evidence points to the MHC region as potentially a vital aspect of etiopathogenesis in psychopathology. The MHC region harbors the strongest genome-wide signal to date for psychotic and affective disorders. It also harbors a known synaptic pruning mechanism as part of the *C4* complex. The strong association with cognitive ability makes MHC a candidate region for further extensive investigation.

Results in the current report demonstrate that it is possible to deconstruct the genetic architecture of psychopathology via pleiotropy with cognitive phenotypes. Nonetheless, the methods reported here are not without limitations. First, we have restricted investigation to GWAS summary statistics derived from studies of European ancestry as a matter of availability. Local genetic correlational methods require relatively large sample sizes for estimation. As more well-powered GWAS become available in other ancestries, it would be necessary to examine if the genetic architecture across cognitive and psychiatric traits would replicate. Relatedly, high-dimensional methodologies such as those reported in the current study tend to be dependent on the statistical power of the trait. As GWAS summary statistics become more powered across the board, genomic clusters not previously discovered may likely emerge. Additionally, it should be noted that the present study utilizes currently available GWAS that are based on common genetic variation and community sampling; in the future, well-powered family-based GWAS may ultimately provide more accurate estimates of genetic effects, including rare variation^51^, independent of “genetic nurture”^52^. Additionally, there may be measured and unmeasured confounders to the GWAS included in our analysis; for example, meta-locus NCF-1 may demonstrate inflated or biased results due to associations with socioeconomic status and should be interpreted with caution. Moreover, CTP and NCF are relatively broadly defined phenotypes that may be further refined by future research, and only capture a portion of the relevant phenotypic space (Fig. S1a); these were selected for our primary analyses because they are relatively well-powered and capture the largest portion of the variance of interest. Finally, high-dimensional data reduction methods, such as UMAP and DBSCAN employed in the present report, can be controversial insofar as they are (by definition) simplifications of data subject to potential over- and under-fitting^53^. Consequently, we employed methods that have been recently demonstrated to be superior to other approaches to reduction of high-dimensional genetics data^54,55^ and we rigorously tested the assumptions of our approaches (see Methods) and their ability to recover structure in ground truth datasets. Our approach to deconstructing the genetic architecture of correlated complex traits via pleiotropy complements other methodologies in the GWAS toolkit, and future work might focus on extending the approach.

To conclude, we have leveraged pleiotropy between cognitive dimensions and psychopathology to dissect biological mechanisms underlying these phenotypes. By compiling local genetic correlations across the genome into “meta-loci,” we identified specific regions of the genome that harbor gene sets representing dissociable biological processes linking psychopathological traits to cognition. Follow-up efforts such as increasing the power of the input trait GWAS and the accuracy of transcriptomic reference panels may increase the resolution of such approaches. Our findings further demonstrate the importance of the recently identified non-cognitive factor to understanding the biology of neuropsychiatric phenotypes; it may be useful for future research to devise a way in which this latent factor could be operationalized and measured tangibly in clinical populations. Results of the current report underscore the need to develop additional statistical methodology to exploit the phenomena of pleiotropy and polygenicity that are characteristics of psychiatric genetics.

## Methods

### Data curation

To thoroughly examine the pleiotropic relationship between cognitive dimensions and psychopathology, we consolidated 19 Cognitive Traits, 17 Psychopathological Traits, Education Attainment, and Socioeconomic Status GWAS summary statistics for the current study. Cognitive Traits included General Cognitive Ability^22^ (indicated as MTAG_GCA in the report), ASSET Discordant^16^, DeMange-Cognition^18^ (shown as GSEM_GCA currently), and DeMange-Non-cognitive Skills^18^ (shown as GSEM_NCS currently). It should be noted that we retained the previously utilized labeling of “general cognitive ability” and “non-cognitive skills” to describe the summary statistics entered in our initial data reduction step, to be consistent with the literature in which the summary statistics were produced. However, for all subsequent analyses, we employ the terms “cognitive task performance” (CTP) and non-cognitive factor (NCF), respectively, which we believe are more accurate descriptively, and reduce opportunities for misunderstanding or misrepresentation of the research.

We also curated GWAS summary statistics for other cognitive traits. These include the GWAS summary statistics obtained via collaboration with Biogen Inc.: Full-Scale IQ, Numeric Reasoning, Verbal Reasoning, General Cognitive Ability (Computed using approaches detailed in Davies et al. (2018)), Pairs Matching, and Reaction Time. Data from Biogen Inc. were the most recent UK Biobank data freeze of cognitive tests. Similar cognitive traits were also available via our collaboration with the Institute of Behavior Genetics (IBG). Both sets of GWAS summary statistics were included for exploration because, for the latter, missing data were imputed for the entire UK Biobank data. (Further details of the imputation method were reported in Hatoum et al.^25^). The following GWAS summary statistics were included from IBG, Executive Function, Digit Symbol, and Trail Making Test. Two sets of Education Attainment GWAS summary statistics were included; the first was reported by Lee et al.^56^, and the second set was from UK Biobank’s latest data freeze. Finally, for follow-up and post-hoc investigation, GWAS summary statistics for the Townsend Deprivation Index were also included.

GWAS summary statistics for psychopathological traits included Attention-Deficit/Hyperactivity Disorder^40^, Anorexia Nervosa^50^, Generalized Anxiety Disorder^57,58^, Bipolar Disorder^59^, Insomnia^60^, Tourette’s syndrome^61^, Autism Spectrum Disorder^62^, Major Depressive Disorder^44,63^, Post-Traumatic Stress Disorder, and Schizophrenia^43^. Notably, two Mood disorder definitions were included. The first was reported by Howard et al.^63^, and the second was obtained via collaboration with the Million Veterans Project^44^. The rationale for including both sets of Major Depressive Disorder GWAS was that the sample combinations were slightly different—the latter including data from the FinnGen study^44^. Similarly, for Generalized Anxiety Disorder, GWAS summary statistics from ANGST consortium^57^ and the Million Veterans Project^58^ were included. Over and above psychiatric traits, several personality dimensions (i.e., Neuroticism, Depressive-Affect, and Worry)^42^ were added to accentuate the analysis. Details and descriptions of GWAS summary statistics are reported in Supplementary Data 1.

### Global genetic correlations (cognitive traits vs. psychopathology)

We carried out global genetic correlation analysis between the set of 19 cognitive traits and 17 psychiatric traits via LD score regression^23,24^ implemented in Genomic SEM^11^. Pairwise LD score regression was carried out via the genetic correlation matrix wrapper function found in the GenomicSEM::ldsc(), stand=TRUE function (GenomicSEM version 0.0.2, https://github.com/GenomicSEM/GenomicSEM). The global genetic correlation matrix was reported in Supplementary Data 2a. GenomicSEM performs initial data alignment to the HAPMAP3 SNPs and conducts pairwise LD score regression with each pair of input phenotypes for the estimation of global genetic correlations. The global genetic correlations between all cognitive and psychopathological traits were organized into a 19 × 17 matrix and subsequently used as the input for principal components analysis (PCA) and clustering analyses reported subsequently. The first two principal components were extracted from the 19 × 17 matrix representing the relationship between each psychopathology trait and the top two cognitive dimensions. The loading of each cognitive trait on the principal component was estimated by performing bivariate Pearson correlation between the genetic correlation profile of each cognitive trait and each principal component (columns U and V in Supplementary Data 2a). The dissimilarity matrix estimated based on Euclidean distance from the global genetic correlation matrix was used to generate the partitioned *k*-medoid clusters. The clustering procedures were carried out using fviz_cluster() in R as part of the “factoextra” package (https://www.rdocumentation.org/packages/factoextra/versions/1.0.3).

### Cluster analyses for the global genetic correlation matrix

Global genetic correlation analysis was first read into R as matrix *x*—from which we proceeded to estimate the Euclidean distance matrix. We selected two broad categories of clustering methodology that had been widely reported in academic literature: (i) partitioning methods; (ii) hierarchical clustering methods. Within partitioning clustering methodology, we estimated fit statistics for *k*-means and *k*-medoid clustering. Moreover, within hierarchical clustering methodology, we calculated fit statistics for agglomerative and divisive clustering. Initial clustering analyses were carried out via the FactoMineR and FactoExtra R packages (version 1.07.999, Le et al.^64^). The strategy for the clustering analysis was as follows—we started with a 2-cluster solution, and gradually increased the number of clusters until any of the methods reached a singleton cluster. Across methodologies, a 7-cluster solution resulted in a singleton cluster, hence we set the maximum number of clusters to 6 and the minimum number of clusters 2. We tested cluster solutions starting from *k* = *2* and increased the number of cluster solutions until one of the four methods yielded a cluster with a single data point. The cluster prior to that was then designated as *k*_max_. In this case, the maximum number of clusters was defined as *k*_max_ = *6*. Fit statistics for each set of cluster analyses were estimated using the cqcluster.stats and cluster.boot() modules from the fpc R package (version 2.2-9, Akhanli and Hennig^65^). The fit statistics used in the current report were extensively discussed by Akhanli and Hennig. For straightforward interpretation of the fit statistics, we scaled the fit statistics such that larger metrics always meant better fit. All fit statistics were summed, based on previously established approaches^9^, to get overall fit statistics for the cluster solutions. A five-cluster solution computed by *k*-means and *k*-medoids appeared to have comparable fit statistics. However, *k*-medoid cluster appeared to have slightly better stability after bootstrapping compared to the *k*-means solution. The cluster analysis results allowed further annotation of the genetic correlation matrix (see Supplementary Fig. 1 and Supplementary Data 2c).

After closely reviewing the global genetic correlation profiles for cognitive features and psychopathological traits described above, CTP and NCF emerged as two candidate traits that were well differentiated across clusters of psychopathological traits. Partitioning cluster analysis (*k*-medoids and *k*-means) and hierarchical clustering were employed to examine the latent genome-wide genetic architecture of psychopathological traits in relation to cognitive features.

### GWAS-by-subtraction: defining the non-cognitive factor

In addition to GWAS summary statistics obtained from publicly available repositories, and/or obtained from closed access datasets, we generated new summary statistics for the “Non-Cognitive Factor” (NCF) via the GWAS-by-subtraction steps reported by Demange et al.^18^ described at https://rpubs.com/MichelNivard/565885. The input GWAS summary statistics differed slightly from the initial report by Demange et al.^18^ We used the publicly available Lee et al.^56^ Education Attainment GWAS summary statistics (without the 23andMe data) and the most powered cognitive task performance GWAS (Lam et al.^22^). This was recommended by the original authors where new information should be added to aid in the definition of the non-cognitive factor. We also note that that the GWAS summary statistics that were included in GWAS-by-subtraction procedures were first quality controlled by earlier summary statistics QC procedures described above. As a sanity check to ensure that GWAS summary statistics were similar to those reported by Demange and colleagues^18^, we carried out LD score regression^23^ on the extracted latent factor scores compared to those that were reported on previously. The results indicate that the genetic correlation between the newly estimated summary statistics and those previously reported by Demange and colleagues^18^ were the same (*R*_g_ = 1). Nevertheless, we noted that the power of the non-cognitive factor was slightly reduced. This could be related to two concurrent reasons. First, Demange and colleagues utilized data from Education Attainment that included the 23andMe data, which was larger. Second, the GWAS summary statistics used earlier was a less powered version for cognitive task performanceat 257 K individuals, compared to that in the current report at 373k estimated sample size. Nonetheless, because the primary objectives of the present report were not loci discovery at the level of GWAS p-values, we made no further loci-based comparisons with the earlier study^18^.

### Local genetic-correlation analysis

Local genetic correlations were carried out via ρ-HESS, based on analytic steps described in https://huwenboshi.github.io/hess/local_rhog/. Local genetic correlations were computed on 2353 LD-independent regions across the genome. The LD independent regions were calculated via LD detect^66^ using variants with minor allele frequencies greater than 0.05. Further details of the estimation of LD independent regions were previously reported^67^. Local genetic correlations were carried out with cognitive task performance and, separately, the non-cognitive factor, with each of the 17 psychopathological traits selected for the current report (ρ-HESS version 0.5.4). For the present report, we wrote a wrapper for ρ-HESS that produces a series of helper scripts that allowed us to scale the analysis. The wrapper scripts could be found at https://github.com/maxzylam/rho-HESS-wrapper.

### ρ-HESS technical results and benchmarks

Heritability of LD independent regions for cognitive task performance (CTP) and the non-cognitive factor (NCF) was reported in Supplementary Data 3. We noted that the summed heritability of each of the cognitive dimensions was consistent with those estimated by global genetic heritability. Summed heritability was calculated by taking the total heritability across all LD independent regions. Local genetic correlations were represented in covariances and *Z*-scores by ρ-HESS. We chose to use the standardized *Z*-scores for downstream analysis to standardize the scaling of the effect sizes. Also, scaled scores tend to be preferred for cluster analyses. The results of CTP and NCF were reported in Supplementary Data 4 and Supplementary Data 5.

The heritability estimates for CTP and NCF summary statistics were compared with what is currently reported in the literature, as well as across LDSC and ρ-HESS.

### Cognitive task performance

Reported by Davies et al.^68^: “The report included all common SNPs using GCTA-GREML in four of the largest individual samples: English Longitudinal Study of Ageing (ELSA: *N*  =  6661, *h*^2^ =  0.12, SE  =  0.06), Understanding Society (*N*  =  7841, *h*^2^ =  0.17, SE  =  0.04), UK Biobank Assessment Center (*N*  =  86,010, *h*^2^ =  0.25, SE  =  0.006), and Generation Scotland (*N*  =  6507, *h*^2^ =  0.20, SE  =  0.05).”

Reported by Savage et al.^69^: “SNP heritability estimated for the entire sample *h*^2^_SNP_ was 0.19 (SE = 0.01) estimated by LDSC.”

#### Estimation of heritability within current study

$${h}_{{{{{{\mathrm{CTP}}}}}}-{{{{{\mathrm{LDSC}}}}}}}^{2}=0.154\,(0.0059)$$

$${h}_{{{{{{\mathrm{CTP}}}}}}-{{{{{\mathrm{HESS}}}}}}}^{2}=0.231\,(1.11{{{{{\rm{e}}}}}}-5)$$

### Non-cognitive factor

Reported by Demange et al.^18^: “*λ*_NonCog-EA_ = 0.2565 (Genomic SEM).”

#### Estimation of heritability within current study

$${\lambda }_{{{{{{\mathrm{NonCog}}}}}}-{{{{{\mathrm{EA}}}}}}}=0.230\,({{{{{\rm{Genomic}}}}}}\,{{{{{\rm{SEM}}}}}})$$

$${h}_{{{{{{\mathrm{NCF}}}}}}-{{{{{\mathrm{LDSC}}}}}}}^{2}=0.204\,(0.0105)$$

$${h}_{{{{{{\mathrm{NCF}}}}}}-{{{{{\mathrm{HESS}}}}}}}^{2}=0.310\,(6.20{{{{{\rm{e}}}}}}-5)$$

Both CTP and NCF are still in the range of what is reported in the literature. It is notable that ρ-HESS is estimating heritability slightly higher than LDSC. This appears consistent between CTP and NCF. The explanation for that is the additional 10% of heritability is likely related to ρ-HESS using genome-wide summary statistics rather than the 1.2 million HAPMAP3 SNPs that LDSC uses.

To examine if there was concordance between LDSC and ρ-HESS, we compared global genetic correlations estimated by both methods. Default parameters were carried out on LDSC, as part of standard procedures. Summary statistics were pruned to 1.2 million HAPMAP3 SNPs for global genetic correlation estimations for LDSC. Bivariate genetic correlations were carried out for CTP and NCF vs. each of the psychopathological traits included in the current study. To estimate global genetic correlation via ρ-HESS, we summed the estimated covariances across all LD-independent regions in the genome and the estimated heritability for either cognitive dimension (CTP/NCF) and psychopathological trait. Genetic correlations were estimated in the following manner

$${R}_{g-{{{{{\mathrm{HESS}}}}}}}=\sqrt{\frac{\sum ({{{{{\mathrm{Co}}}}}}{{{{{{\mathrm{v}}}}}}}_{j})}{\sum (h\frac{2}{ja}*h\frac{2}{jb})}}$$

where *j* is a given set of LD independent regions, Cov represents the covariance at each LD independent region and, ${h}_{j}^{2}$ is the heritability of *j* regions; *a* represents cognitive dimensions, either CTP or NCF, and *b* represents psychopathological trait.

A scatterplot was constructed to visualize the concordance between LDSC and ρ-HESS (Supplementary Fig. 2).

Manhattan plots for the local genetic correlation output (in *Z*-scores) were visualized in several ways—first, each local genetic correlation on each LD segment was visualized based on genomic coordinates. Next to show the range of local genetic correlations we aligned the LD segments from lowest local *R*_g_ to highest *R*_g_ (See Supplementary Fig. 3). Owing to the complex LD structure harbored within the MHC region, the region was excluded from subsequent cluster analysis. However, for completeness of the results, we further analyzed the local genetic correlation within the MHC region to understand how this region might feature in the pleiotropic relationship between cognitive traits and psychopathology. The MHC region was visualized similarly with the rest of the local genetic correlations segments Supplementary Fig. 4). There were 23 LD-independent segments that were part of the MHC region.

### Significant local genetic correlations of psychopathology with CTP and NCF

To further investigate local genetic correlations between cognitive dimensions (CTP/NCF) and psychopathological traits, we flagged LD-independent regions showing strong local genetic correlation *Z*-scores (|Z | > 4, correcting for multiple testing in 2353 LD independent regions, assuming *Z* = 1.96 represents *p* = 0.05 (see Supplementary Data 6). 88 LD-independent regions showed strong local genetic correlations between CTP and schizophrenia; 27 LD-independent regions showed strong local genetic correlations between NCF and schizophrenia. 13 LD-independent regions showed strong correlations with affective traits and CTP, and 27 LD-independent regions showed strong correlations with affective traits and NCF.

### Local genetic correlations for cognitive traits and socioeconomic status (SES)

The relationship between CTP and NCF with SES was further investigated at the local genetic correlation level. Like local genetic correlations for psychopathological traits, we included SES defined by the Townsend Deprivation Index available as a phenotype within the UK biobank. These results were later stratified by meta-loci. Results will be further discussed in subsequent sections (see Supplementary Data 7).

### Conceptual overview of the density-based spatial clustering of applications with noise (DBSCAN) clustering algorithm in establishing genomic meta-loci

Density-based spatial clustering of applications with noise (DBSCAN) procedure^70^ is a sequential clustering methodology that could be applied to high-dimensional data that also tends to be noisy. The DBSCAN algorithm works on data clustering based on a given radius and minimum points per cluster parameter. For each data point, in this case, for each LD-independent region, DBSCAN estimates the distance relative to all 2330 LD-independent regions (without the MHC region). If the distance is less than or equal to the epsilon, then the LD-independent region would be marked as a neighbor of x. If the LD-independent region gets a neighboring count greater than or equal to the minimum points per cluster, DBSCAN marks the region as a core point. For each core point, if not already assigned to a cluster (meta-locus), the algorithm then creates a new cluster (meta-locus). DBSCAN then recursively finds all neighboring points and assign them to the same cluster (meta-locus) as the core point. These steps are iterated until all LD-independent regions were either assigned cluster membership (or all points assigned to a meta-locus) or indicated as an outlier. For further details, see https://towardsdatascience.com/k-means-vs-dbscan-clustering-49f8e627de27, and http://www.sthda.com/english/wiki/wiki.php?id_contents=7940.

### Approach for meta-loci identification

To establish the meta-loci from a 2353 × 17 matrix, we carried out a series of procedures to uncover latent cluster structure of the local genetic correlations across CTP and NCF with psychopathology. Owing to DBSCAN’s nature of being a fully unsupervised sequential clustering algorithm, it would be necessary to carry some form of data reduction procedure prior to the clustering step. There were several methods that were available for data reduction, e.g., Principal Components Analysis (PCA), or UMAP. We decided that UMAP dimensionality reduction is preferred as it permits non-linear combination of dimensions, that might allow more efficient data reduction for the large high-dimensional data matrix that we are attempting to decompose. Unless otherwise stated, most of the procedures described in the current section utilizes the CTP local genetic correlation matrix—for several reasons (i) The CTP matrix is more powered than NCF (ii) interpretation of CTP, i.e., cognitive test performance, has been extensively discussed in literature elsewhere.

### Step 1: Identify if a latent data structure exists for cluster analysis

Prior to carrying dimensional reduction and clustering analysis, it would be necessary to ascertain if a latent data structure exists. This is typically established using the Hopkins test statistic^71^—known to be a fair estimator for randomness in a dataset^72^. An estimated value close to 0.90 is thought to indicate a high probability of the presence of clustered data structure, whereas an estimated value close to 0.50 suggests that the data is random. We applied the Hopkins statistics to both CTP and NCF, which returned a value of 0.92 for both datasets, respectively, suggesting that the data is highly clustered. To ascertain that the Hopkins statistics is in fact measuring the latent data structure, we carried out random shuffling of the CTP matrix. The shuffle procedure was carried out by the sample() module available in R. We shuffled the CTP matrix 20 times and evaluated each Hopkins statistics—the average Hopkins statistic was 0.564 (se = 0.00128) indicated that the shuffled data is likely random.

### Step 2: Identify dimensional reduction strategy for UMAP

Data reduction on the local genetic correlations between the CTP and NCF dimensions and psychopathological traits was carried out via Uniform Manifold Approximation and Projection for Dimension Reduction (UMAP, McInnes, et al.^73^). The method was implemented in R 3.6.3 via the uwot package (version 0.1.10). UMAP had previously been demonstrated to be superior in retaining data structure in comparison with other similar methods such as t-SNE^73^. We required the number of nearest neighbors to be five and estimated the minimum spread value for the 2353 LD-independent regions to be ${{{{{{\mathrm{mindist}}}}}}}=\frac{1}{\sqrt{{n\; {{{{{\mathrm{regions}}}}}}}}}$. We also assumed that ~70% of the manifolds are likely to show local connections. One of the issues with utilizing UMAP as a dimension reduction strategy is that there Is a stochastic element built into the algorithm. As such, though UMAP is effective in capturing the latent data structure of the local genetic correlation matrix, minor perturbations of the actual coordinates would invariably affect a sequential cluster algorithm, where the actual coordinate of the data point matters. To evaluate the stability of the UMAP solutions, we took into account Trustworthiness and Continuity measures^74^. Both Trustworthiness and Continuity are indexes that range from 0 to 1 and measure the degree to which the original data structure was retained after dimensional reduction. Trustworthiness and Continuity close to 0.9 is considered well fitting.

For the current study, we examined the best approach to obtain UMAP dimensions that allows data reduction and at the same time retains data structure. First, we attempted to extract 3-dimensional solutions for UMAP, compared to the more standard practice of using the default of 2-dimensional UMAP solution. 20 UMAP models of 3 and 2-dimensional solutions were generated. To constrain the stochastic nature of UMAP we constrained the random number generator using a consistent 20 element vector of seeds. Across 20 solutions, 2-dimensional UMAP solutions are much more consistent for UMAP1 (Pearson *r*_average_ = 0.94) and UMAP2 (Pearson *r*_average_ = 0.87) as compared to 3-dimensional solutions, which appeared poorly replicated for each consecutive model extracted: UMAP1 (Pearson *r*_average_ = 0.55), UMAP2 (Pearson *r*_average_ = 0.51) and UMAP3 (Pearson *r*_average_ = 0.55). To investigate if UMAP procedures had indeed retained the original data structure we simply took the product of trustworthiness and continuity—any value that is close to 1 would indicate high data structure retention. The average of all 20 models generated for the 2-dimensional and 3-dimensional UMAP models was 0.851 and 0.866, respectively. These initial numbers indicate that the 3-dimensional model allows for the original data structure to be captured slightly better than the 2-dimensional model. However, given that the Pearson *r*_average_ for the replications of the 3-dimensional solutions was substantially lower than that observed for the 2-dimensional solutions, the small increase in variance explained is accompanied by a large degree of additional noise entering the 3-dimensional solutions. Therefore, considering the subsequent intention to enter the reduced data as stable cluster features, the data indicate that a 2-dimensional solution is more appropriate for the objectives in the current report.

### Step 3: Generating cluster features for DBSCAN cluster analysis

In the previous step, we found that 2-dimensional UMAP solutions were stable, with each UMAP model appropriately retaining the original data structure. Nonetheless, due to DBSCAN’s sequential cluster approach, the procedure is highly sensitive to the actual coordinate of a given data point. As such DBSCAN reveals a slightly different interpretation of the data structure, depending on the UMAP features entered. To maximize the stability of the DBSCAN procedures we increased the UMAP dimensions—not within each UMAP model (i.e., increasing extracted dimensions); rather, we increased the number of 2-dimensional UMAP models. However, increasing UMAP models infinitely is likely to introduce too much noise, and the data structure could no longer be parsed. The challenge is to identify the best signal to noise ratio for DBSCAN procedures. As such, we extracted 2-dimension * 5 model UMAP (10 dimensions), 2d * 10 model UMAP (20 dimensions) and 2d * 20 model UMAP (40 dimensions) as input cluster features. This would allow us to evaluate the silhouette score as a function of the number of UMAP dimensions entered as features, to optimize the signal-to-noise ratio for the identification of meaningful clusters by DBSCAN.

### Step 4: Setting up DBSCAN for identification of meta-loci

DBSCAN was carried out for CTP and NCF separately. As previously indicated, the MHC region was excluded from these analyses. However, due to reasons that were discussed in Steps 2 and 3, we attempted to evaluate the methodology to which DBSCAN procedures might yield the most robust and accurate cluster solutions. To achieve this, we relied on the Iris Dataset^75^. The Iris dataset contains 4 features and the given identity of the flowers. The Iris dataset had originally been utilized as the ground truth dataset for linear discriminant function analysis^75^.

DBSCAN was set up such that nearest neighbors were set to minimums of 5. To find the optimal set of meta-loci represented by the data, we first computed the *k*-nearest neighbor distance for all points using a kd-tree. The “elbow” that emerges plotting nearest distance and distance between points, would correspond with the most appropriate eps value for DBSCAN procedures. To further optimize DBSCAN, we increased eps stepwise using the follow approach:

$${N}_{{{{{\rm{eps}}}}}}+{N}_{{{{{\rm{eps}}}}}}*{j},$$

where *N*_eps_ is the epsilon value derived from visualizing the “elbow” in the previous step and *j* ranges from 1 to 5. In most cases, DBSCAN returns a single cluster solution at *j* = 5. For purpose of the current report, we increased eps using the above heuristic mainly to stick to general fitness of the model rather than carry out minute fine-tuning of the model as a form of standardization. Cluster silhouette scores *S*_i_ were utilized to evaluate the appropriateness of DBSCAN solutions. *S*_i_ ranges from −1 to +1^76^. If the silhouette score is closed to 1, it would suggest that the clusters are well identified. A score that is close to 0 would indicate clustering that are indistinguishable, and a negative score would suggest misclassification in the cluster solutions. For purpose of the current report, we considered median, 75th percentile and maximum silhouette scores for all clusters identified in each DBSCAN cluster model. Any models that showed negative silhouette scores were not further considered. Using this approach, we attempted to pick the “best-fitting” DBSCAN model for further investigation.

In the initial phase, we applied UMAP data reduction and DBSCAN procedures to the Iris dataset to examine if the approach resulted in the misclassification of the ground truth categories. DBSCAN procedures showed that a 5 model UMAP gave the best-fitting clustering solutions recovering all original categories with no misclassifications. Nonetheless, DBSCAN also appeared to have identified two additional sub-clusters within the Iris dataset. Although it was unclear if there was in existence additional sub-clusters of flowers, it was reasonable to note that DBSCAN was designed to optimize localized cluster patterns. The approach using a combination of UMAP and DBSCAN procedures were sufficient to recover broad cluster patterns within the data. The preliminary data investigation is summarized in Supplementary Fig. 5.

### Step 5. Procedures for identification of meta-loci in CTP and NCF

For the identification of meta-loci within CTP and NCF, we carried out procedures indicated in the previous step. First, reducing local genetic correlation matrix 2330 (LD independent segments, MHC removed) * 17 psychopathological traits for CTP and NCF, respectively, via UMAP data reduction. 5, 10, and 20 UMAP model solutions were extracted as previously described. These were then entered into DBSCAN as clustering features. For each DBSCAN cluster extraction, we note the number of UMAP dimensions, epsilon value, unclassifiable meta-loci, number of DBSCAN clusters extracted, the median, mean, 75th percentile, and maximum silhouette score. All solutions with negative median silhouette scores were not further considered. To select the final DBSCAN model, we considered the number of unclassifiable LD segments, the number of classified LD segments, and silhouette scores. Aside from having the “best-fit” model, we also considered models that preserved more information for downstream analyses. We report DBSCAN metrics in Supplementary Data 8a for CTP and Supplementary Data 9a for NCF. Finally, to recover global cluster patterns, we subjected the DBSCAN localized cluster patterns and the input UMAP model, to hierarchical cluster analysis. Software packages for hierarchical analysis were similar to those used for global genetic correlation analysis described in the earlier “Cluster analyses for the global genetic correlation matrix” section. To standardize hierarchical cluster solutions, we compared the fit indices for agglomerative hierarchical clustering solutions in Supplementary Data 8b and Supplementary Data 9b for 5, 10, 15, 25, and 30 global clusters. As there are multiple fit indices available for each hierarchical cluster solution, the final hierarchical cluster solution was selected based on the criteria of having the most fit indices being in the top 3 ranked across hierarchical cluster models. Descriptive statistics for the final model were reported in Fig. 2, Supplementary Data 8c for CTP and Supplementary Data 9c for NCF.

The expectation was that DBSCAN procedures clustered LD-independent regions with distinct local genetic correlation profiles and localized by LD. To further examine if each LD-independent region was indeed clustered via local genetic correlation patterns, we visualized each meta-locus for CTP and NCF on genome-wide karyogram (see Fig. 2). The visualization showed that regional distributions were well distributed across the genome and did not appear to be varying by LD or localized effects in the genome. These results increased our confidence that regional local genetic correlations were likely driving the DBSCAN clustering rather than LD patterning across the genome. To further understand the nature of local genetic correlation distributions underlying each meta-locus, distributional patterns of the local genetic correlations with each set of LD-independent regions defined by the meta-loci in CTP or NCF were visualized. First, we re-sorted the Manhattan plots showing the local genetic correlation patterns by coordinates, stratifying them by each meta-locus. Within each meta-locus, we visualized the distributional patterns for local genetic correlations for each cognition-psychopathological trait pair (doing the same for both CTP and NCF) (see Fig. 3, Supplementary Fig. 6, and Supplementary Fig. 7).

We also carried out additional local genetic correlations to examine the potential effect of SES on CTP and NCF. The local genetic correlation distributions across LD segments were stratified by meta-loci and reported in Supplementary Fig. 8.

### Functional annotation and gene prioritization

Functional annotation and gene prioritization was carried out only for GWAS summary statistics indexing cognitive task performance and the non-cognitive factor. The rationale for carrying out gene prioritization in this manner was such that we could have a signal that is cognitive-centric rather than driven by factors that might be specific to or related to the psychopathological conditions investigated. Both CTP and NCF were estimated from the general population, hence, less likely to be influenced by extraneous clinical factors (e.g., illness trajectories and medication) that might have been uniquely driven by the psychopathological condition. Gene prioritization approaches used are considered standard in GWAS downstream analysis. These could be categorized into gene-based genome-wide association approaches (MAGMA and POPs), and transcriptome-wide association approaches (S-PrediXcan, SMR/HEIDI, and FOCUS transcriptome-based finemapping). In the current report, results of the downstream analysis were additionally assigned to the respective CTP or NCF meta-loci.

### MAGMA gene-based genome-wide association (GBGWA) and polygenic priority score (PoPs)

MAGMA gene-based genome-wide association analysis^30^ was carried out for CTP and NCF. GWAS SNP-based summary statistics were used as input data for the MAGMA GBGWA analysis. Gene definitions based on b37 were utilized (see https://ctg.cncr.nl/software/magma). Note that for the current analysis, the latest version of MAGMA v1.08 was utilized using SNP-Wide Mean mode. Further data analysis steps could be found on the provided website. Results of GBGWA are reported in Supplementary Data 10. PoPs is a gene prioritization method that leverages genome-wide signal from GWAS summary statistics and incorporates data from an extensive set of public bulk and single-cell expression datasets, curated biological pathways, and predicted protein-protein interactions. Methodological details of PoPs were previously described by Weeks and colleagues^34^. Data analytic steps are provided at (https://github.com/FinucaneLab/pops). PoPs leverages 57,543 gene features for prioritization. 40,546 features were derived from gene expression data, 8718 features extracted from protein-protein interaction network, and 8479 features based on pathway membership. PoPs was carried out for both CTP and NCF.

### Summary statistics Mendelian Randomization and heterogeneity in dependent instruments analysis

SMR allows the indirect mediating effect of gene expression to be incorporated into the SNP/Variant phenotype effects, while HEIDI allows potential heterogeneity of the mediating effect caused by linkage to be also modeled in the analysis^77^. For the current report, we used eQTL annotations from the Brain e-META database (meta-analysis of GTEx, Common Mind Consortium, and ROSMAP brain-eQTL data), see Supplementary Data 11a; and the PsychENCODE data. Two versions of the PsychENCODE data were used: HCP (Supplementary Data 11b) and PEER (Supplementary Data 11c) adjusted. The union of results from all three annotation databases was considered to minimize prioritization related to methodological variance. Note that because HEIDI assumes that genes with significant results were less likely to have eQTL mediating the SNP-phenotype effect due to linkage, we inverted the significant effects during the gene prioritization procedures.

### Summary statistics PrediXcan (S-PrediXcan) transcriptome-wide analysis

S-PrediXcan, (formerly known as MetaXcan) was carried out to leverage eQTL data for gene prioritization (Oct 16, 2020, version). Details of the S-PrediXcan methodology are now well established and can be found in the report by Barbeira and colleagues^78^. For the current analysis, we leveraged the latest GTEx8^26^ eQTL database. However, to allow more focused functional annotations and gene prioritization processes, we only selected eQTL data for neural tissues. These include Anterior Cingulate Cortex, Amygdala, Caudate—Basal Ganglia, Cerebellum, Cerebellar Hemisphere, Cortex, Frontal Cortex, Hippocampus, Hypothalamus, Nucleus Accumbens, Putamen—Basal Ganglia, Spinal Cord, and Substantia Nigra (Supplementary Data 12a-m).

### FOCUS transcriptome finemapping analysis

The FOCUS^79^ (Fine-mapping Of CaUsal gene Sets) transcriptome finemapping analysis was designed to identify credible genes based on eQTL annotations, leveraging state-of-art GWAS and transcriptomic-based statistical finemapping approaches. For our analysis, we included all genes that were identified as credible genes as part of gene prioritization procedures. FOCUS finemapping procedures and eQTL annotations are available at https://github.com/bogdanlab/focus. To keep consistent with the eQTL annotations of other transcriptomic association methods that were used in the current report, we only selected finemapping results based on brain tissue expression of the Anterior Cingulate Cortex, Amygdala, Caudate—Basal Ganglia, Cerebellum, Cerebellar Hemisphere, Cortex, Frontal Cortex, Hippocampus, Hypothalamus, Nucleus Accumbens, Putamen—Basal Ganglia, Spinal Cord, and Substantia Nigra. We note that in the case of FOCUS transcriptome finemapping, the software outputs only the credible genes per eQTL (Supplementary Data 13a, b). All credible genes were included in the final gene list for prioritization.

### Gene ranking and prioritization

To rank and select pertinent genes for downstream analysis, we carried out a series of gene ranking procedures. This was achieved by taking the 50th percentile cutoff for MAGMA GBGWA, PoPs gene scores, transcriptome association methods, and taking the union of gene lists emerging from these methods with credible genes identified by FOCUS transcriptomic finemapping (Supplementary Data 14a–c). For transcriptomic association methods that utilized more than one eQTL annotation database for prioritization, we used the average gene rank across annotations. The 50th percentile cutoff and corresponding gene rank for MAGMA GBGWA was 8867_CTP_/8870_NCF_, PoPs was 9164_CTP_/9170_NCF,_ and TWAS 3806_CTP_/3771_NCF_ (see Supplementary Data 14a, b). We then attempted to annotate genes that have been prioritized via the HUGO gene annotation database^80^, at this stage, each gene was assigned to their respective meta-locus based on their genomic coordinates (Supplementary Data 14c). To prepare for downstream gene set analysis, we carried out inverse rank score transformation of PoP score on genes that were selected from the 50th percentile from gene prioritization and transcriptomic analyses. The inverse rank score ranges from 0 to 1, and is derived as follows:

$$1-{{{{{\rm{ran}}}}}}{{{{{{\rm{k}}}}}}}_{n}/{{{{{\rm{ran}}}}}}{{{{{{\rm{k}}}}}}}_{{{{{{\rm{i}}}}}}}$$

where rank_*n*_ is the rank for a given gene, and rank_i_ is the highest-ranking gene for a given trait. The resultant score would reflect 0 as the lowest ranking gene, and 1 as the highest-ranking gene. Inverse rank scores are provided in Supplementary Data 15.

### Gene-set analyses

To further annotate putative biological mechanisms and processes underlying each meta-locus for either cognitive dimension (CTP/NCF), we carried out gene set analysis on gene lists assigned to each meta-locus. Three separate gene set analysis approaches were then applied to the remaining, filtered gene list for each meta-locus (i) Broad Institute Gene-Set Enrichment Analysis (GSEA^31,81^) (ii) WebGestalt ORA^32^ (iii) FUMA GENE2FUNC^33^. GO ontologies within the Molecular Signature Database 7.2 (Biological Processes, Molecular Function and Cellular Component^36,82^) were used as gene-set analysis annotations.

Of the three gene set analysis methods, GSEA was arguably the most robust. GSEA first walks down the ranked list of genes, increasing a running-sum statistic when a gene is in the gene set and decreasing it when it is not. The enrichment score is the maximum deviation from zero encountered during that walk. Details of the GSEA methodology has been reported elsewhere^31^. GSEA prioritizes genes via the following heuristic: given a defined gene list *L*, GSEA determines if the genes are randomly distributed throughout pathway *S* or primarily found at the distributional tails. This is achieved via estimating an enrichment score based on any given metric that represents correlation with a given phenotype. In the current study, the selected phenotypes were CTP and NCF. The gene-phenotype correlation was denoted in the current study via gene scores estimated by PoPs, where the higher the metric, the more likely the gene was associated with CTP or NCF. However, it is necessary to note that PoPs gene score is unidirectional, unlike gene expression. For gene expression, strong effect sizes in either direction represent strong gene-phenotype associations. However, for PoPs gene score, negative scores do not denote stronger associations with the phenotype. Rather, negative scores denote poor gene-phenotype associations. Hence, for the PoPs gene score to be incorporated into the GSEA gene scoring algorithm, we carried inverse rank scoring to scale the PoPs gene score to represent the unidirectionality of the metric, such that 0 represented poor gene-phenotype association and 1 represented the strongest gene-phenotype relationship. The procedure had been described in the earlier sections.

GSEA allows permutation testing for the selection of gene sets, which in this case was set to *n* = 1000 permutations. GSEA analyses were based on the “preranked” procedure, where enrichment scores were normalized. Default filter parameters for minimum (>15) and maximum (<500) gene set size were used. MSigDb version 7.2 gene set definitions^36^ for Gene Ontology were used as indicated above. The gene list for each meta-locus was ordered by PoPs gene score. GSEA then compares each gene within the meta-locus against each gene sequentially within pre-defined pathway genes. If a gene within a given meta-locus and pathway matches, the gene score is summed. If the gene is not represented in the pathway, the gene score is subtracted. The running hypothesis is that if a list of genes is random, the enrichment score would be likely to tend towards the null. Whereas if a list of genes is well represented within a given pathway, there would be a significant deviation from the null. The null enrichment score was estimated by randomly ordering the association metric with the gene list and re-computing the enrichment score. This was repeated 1000 times to get a null distribution. Significance testing was carried out by testing if a given enrichment score for a particular gene set significantly deviated from its null distribution. In addition to the enrichment score for each gene set, it was possible to identify “driver genes” via GSEA. Driver genes are the core of a gene set accounting for the enrichment signal. Driver genes could be identified as those whose running-sum statistic deviates for a given gene set, farthest from the null. GSEA gene sets that are FDR < 0.05 were considered significant.

This is followed by WebGestalt ORA^83^. WebGestalt uses a more specifically curated “noRedundant” set of Gene Ontologies based on the 2017 data freeze of the MSigDb. In addition, the method relaxes gene set sizes to permit minimum (>5) and maximum (<2000). We set significance to the top 50 gene sets to be extracted for WebGestalt. In addition to the Gene Ontologies we also entered DrugBank (https://go.drugbank.com/) annotations to further allow us to understand how biological mechanism within each meta-loci might relate to known genes that are targets of pharmacological compounds. It would also be necessary to note that due to the “noRedundant” feature of Webgestalt’s ORA analysis, we considered gene sets with FDR < 0.1 as supporting evidence—if a gene set was previously identified as significant within GSEA, and FUMA::GENE2FUNC, then a nominal significant p-value for WebGestalt would be taken into consideration. FUMA GENE2FUNC uses a hypergeometric approach to gene selection, which relies only on the overrepresentation of gene symbols for the identification of gene sets. For FUMA GENE2FUNC we set a minimum of 3 genes per gene set and FDR < 0.05 for a gene set to be significant.

To select candidate gene sets for each meta-locus, we required a “consensus” approach. As GSEA is a more robust approach, the results were considered primary. Webgestalt ORA and FUMA::GENE2FUNC approaches were considered secondary evidence for gene set association. For a gene to be included for further consideration it had to be significant for GSEA and at least supported by one other method. Using GSEA as a strategy to identify driver genes for each gene set identified per meta-locus, coupled with the requirement for multiple gene set analysis to identify converging gene set, over and above earlier MAGMA GBGWA and transcriptomic methods, we were able to identify a very specific list of genes for each meta-locus that were putatively responsible for biological mechanisms that might be subserved within each CTP or NCF meta-locus.

We reported the gene-set analysis Gene Ontology (Biological Process, Molecular Function and Cellular Component) results in Supplementary Data 16a, and Drug pathway gene-set analysis results in Supplementary Data 16b.

### BrainSpan spatial-temporal gene expression analysis

As a function of earlier gene set analysis and gene prioritization approaches, we were able to identify that potentially CTP could have been associated with neurodevelopmental mechanisms, while NCF could have been associated with synaptic function. We tested the hypothesis, using driver genes identified by GSEA earlier, that the spatial-temporal gene expression of driver genes indexing neurodevelopmental mechanisms were likely to be significantly expressed prenatally, whereas driver genes that were responsible for the synaptic function would potentially be stable across the lifespan, if not show a preponderance of expression in adulthood.

### BrainSpan data preparation

BrainSpan data was access via https://www.brainspan.org/static/download.html. RNA-Seq Gencode v10 summarized to genes database, that include normalized gene expression was utilized for the analysis. For purposes of data analysis, we recoded the developmental stage into “Weeks” of development so that we could obtain a higher resolution of the spatial-temporal gene expression profile. All post-natal stages were converted to weeks using *Weeks* = Years*52*Weeks* + 37 *Gestational Weeks*, which resulted in the *Weeks* variable ranging from 8 to 2117 weeks. Gene expression for driver genes within the prioritized meta-loci for CTP and NCF were extracted and aggregated by taking the mean expression of all driver genes within a given meta-locus.

### Linear mixed modeling for evaluating longitudinal spatial-temporal gene expression trajectories

To test for gene expression trends over the lifespan for CTP and NCF, and for each meta-locus, we carried out linear mixed modeling using the lme4^84^ and lmeTest^85^. For the overall comparisons of cognitive task performance and the non-cognitive factor, we dummy coded genes falling into each respective category and the dummy coded variable as a *Trait* variable (CTP vs. NCF). Random effect estimator was denoted for individual subjects within the BrainSpan database. As *Weeks* was a variable of interest, we did not covary the analysis for the developmental stage. Sex was included as a covariate. The null model was specified as *Expression* ~ *β*_1_ *Weeks* + *β*_2_ *Sex* + (1|*subject*) and the alternative model to test for the effect of the *trait* was *Expression* ~ *β*_1_ Weeks*Trait + *β*_2_ *Weeks* + *β*_3_ *Trait* + *β*_4_ *Sex* + (1|*subject*). A significant interaction effect in the alternative model would suggest that different profiles of spatial-temporal gene expression across the lifespan were present between CTP and NCF, respectively. Post-hoc analysis was carried out to examine specific meta-loci within CTP and NCF that were driving the interaction effect. The model *Expression*_meta-locus_ ~ *β*_1_ *Weeks* + *β*_2_ *Sex* + (1|*subject*), where *meta-locus* represented each of the prioritized meta-loci, for CTP and NCF, respectively, was used to evaluate if gene-expression within the meta-locus was prenatal, adulthood, or lifetime. The corresponding expectation for each scenario would be a significant positive effect, significant negative effect, and not significant.

Aggregated gene expression trajectories for each meta-locus are displayed in Supplementary Fig. 9. Overall interaction effects of CTP and NCF by time are presented in Fig. 5. Results of linear mixed models examining the significance of longitudinal trajectories are reported in Supplementary Data 17.

### Allen Human Brain Atlas BrainScope visualization

To further understand results from the meta-locus concept, we annotated driver genes being expressed in each meta-locus. This is especially important for any particular gene set that had been identified as significant across multiple meta-loci. For instance, the Axon Development gene set was identified by gene set enrichment analysis at meta-locus 1, 2, 5, 11, 12, 13, and 15 for CTP. Since each driver gene list for meta-loci are mutually exclusive, the results would have indicated that a critical number of genes within each meta-locus would have been identified for the gene set. This is especially true for large neurodevelopmental gene sets.

We utilized gene expression data from the Allen Human Brain Atlas (AHBA^86,87^) available via the BrainScope^88^ web visualizer. Gene expression within the BrainScope visualizer is displayed as red for upregulation and blue for downregulation. Brain regions are displayed on three coronal slices that allows clear visualization of subcortical and cortical regions. Results would allow visualization of putative gene expression networks that might be subserved within each meta-locus. Brain regions with annotated gene expression from BrainScope are displayed in Supplementary Figs. 10–14. Neurodevelopmental gene sets that constitute 6 or more meta-loci were selected for BrainScope/AHBA visualizations.

### Driver gene annotations

Finally, driver genes were annotated with GWAS catalog information and Druggability tier information for the reader’s reference. GWAS catalog (r2022-03-23) was downloaded on March 23, 2022. Druggability tier information is based on Finan et al. (2017)^38^.

### Software utilized

(1) GWAS Summary statistics quality control: SumstatsQC v0.1 (https://github.com/maxzylam/SumstatsQC). (2) Global Genetic Correlations: GenomicSEM version 0.0.2 (https://github.com/GenomicSEM/GenomicSEM); (3) *k*-Medoid clustering and Principal Components Analysis: FactoMineR and FactoExtra R packages (version 1.07.999, Le et al., 2008); fpc R package (version 2.2-9). (4) GWAS-by-Subtraction: GenomicSEM version 0.0.2 (https://github.com/GenomicSEM/GenomicSEM). (5) Local Genetic Correlations: ρ-HESS version 0.5.4 (https://huwenboshi.github.io/hess/local_rhog/); Wrapper script for p-HESS (https://github.com/maxzylam/rho-HESS-wrapper). (6) UMAP/Density-Based Scan: uwot package (version 0.1.10); dbscan package (version 1.1.5) (7) Transcriptome Wide Analysis: MAGMA Gene-Based Genome-Wide Analysis; MAGMA v1.08 (https://ctg.cncr.nl/software/magma); PoPs Gene Polygenic Priority Score-PoPs v0.1 (https://github.com/FinucaneLab/pops); Summary Statistics Mendelian Randomization/HEIDI-SMR/HEIDI version 1.03 (https://cnsgenomics.com/software/smr/#Download); Summary statistics PrediXcan TWAS-SPrediXcan (Oct 16, 2020 version) (https://github.com/hakyimlab/MetaXcan); FOCUS transcriptomic finemapping-FOCUS (Aug 21, 2020 version) (https://github.com/bogdanlab/focus). (7) Gene-Set Analysis: FUMA::GENE2FUNC-FUMA v1.36a https://fuma.ctglab.nl/; WebGestalt-Version 2019 http://www.webgestalt.org/; Gene-Set Enrichment-GSEA 4.10 (https://www.gsea-msigdb.org/gsea/index.jsp); (8) Spatial-Temporal Gene Expression: BrainSpan-RNA-Seq Gencode v10 summarized to genes database https://www.brainspan.org/static/download.html; Linear Mixed Model analysis for BrainSpan Data-lmerTest package (version 3.1.3) (9) General biostatistics/data wrangling: R-statistics version 3.6.3 (10) Allen Human Brain Atlas visualizations: BrainScope https://brainscope.lumc.nl/brainscope.

### Reporting summary

Further information on research design is available in the Nature Research Reporting Summary linked to this article.

## Supplementary information

Supplementary Information

Description of Additional Supplementary Files

Supplementary Data 1

Supplementary Data 2

Supplementary Data 3

Supplementary Data 4

Supplementary Data 5

Supplementary Data 6

Supplementary Data 7

Supplementary Data 8

Supplementary Data 9

Supplementary Data 10

Supplementary Data 11

Supplementary Data 12

Supplementary Data 13

Supplementary Data 14

Supplementary Data 15

Supplementary Data 16

Supplementary Data 17

Supplementary Data 18

Reporting Summary

## Data Availability

(1) The GWAS summary statistics for Cognitive Task Performance and Non-Cognitive Factor data generated in this study are available at https://storage.googleapis.com/broad_institute_mlam/brainstorm-v2-local-gencor-1/03_quality_control_sumstatsqc/07_Data_Release_GWAS_Catalog_01/Lam_et_al_2021_CognitiveTaskPerformance.tsv.gz; https://storage.googleapis.com/broad_institute_mlam/brainstorm-v2-local-gencor-1/03_quality_control_sumstatsqc/07_Data_Release_GWAS_Catalog_01/Lam_et_al_2022_NonCognitiveFactor.tsv.gz; The individual genotype data are protected and are not available due to data privacy laws. The processed individual genotype data can be obtained by contracting respective laboratories that contributed to the data. The meta-data for Cognitive Task Performance and Non-Cognitive Factor GWAS summary statistics generated in this study are provided in Supplementary Data 1. (2) Previously unpublished GWAS data that was closed access to Biogen Inc. would now be made available. GWAS summary statistics for Education Attainment, General Cognitive Ability, Numeric Reasoning, Pairs Matching, Reaction Time, Verbal Reasoning, and Social Deprivation used in this study are available at https://storage.googleapis.com/broad_institute_mlam/brainstorm-v2-local-gencor-1/03_quality_control_sumstatsqc/07_Data_Release_GWAS_Catalog_01/Biogen_2022_Education_Attainment.tsv.gz; https://storage.googleapis.com/broad_institute_mlam/brainstorm-v2-local-gencor-1/03_quality_control_sumstatsqc/07_Data_Release_GWAS_Catalog_01/Biogen_2022_General_Cognitive_Ability.tsv.gz; https://storage.googleapis.com/broad_institute_mlam/brainstorm-v2-local-gencor-1/03_quality_control_sumstatsqc/07_Data_Release_GWAS_Catalog_01/Biogen_2022_Numeric_Reasoning.tsv.gz; https://storage.googleapis.com/broad_institute_mlam/brainstorm-v2-local-gencor-1/03_quality_control_sumstatsqc/07_Data_Release_GWAS_Catalog_01/Biogen_2022_Pairs_Matching.tsv.gz; https://storage.googleapis.com/broad_institute_mlam/brainstorm-v2-local-gencor-1/03_quality_control_sumstatsqc/07_Data_Release_GWAS_Catalog_01/Biogen_2022_Reaction_Time.tsv.gz; https://storage.googleapis.com/broad_institute_mlam/brainstorm-v2-local-gencor-1/03_quality_control_sumstatsqc/07_Data_Release_GWAS_Catalog_01/Biogen_2022_Social_Deprivation.tsv.gz; https://storage.googleapis.com/broad_institute_mlam/brainstorm-v2-local-gencor-1/03_quality_control_sumstatsqc/07_Data_Release_GWAS_Catalog_01/Biogen_2022_Verbal_Reasoning.tsv.gz. The individual genotype data are available under restricted access, access can be obtained by application to the UK Biobank. The meta-data for the cognitive summary statistics are provided Supplementary Data 1. (3) Polygenic Priority Score gene features used in this study are available at https://github.com/FinucaneLab/pops. Results from the Polygenic Priority Score analysis is available in Supplementary Data 10 (4) eQTL annotations from the Brain e-META, PsychENCODE PEER methodology and PsychENCODE HCP methodology are available at the Summary Statistics Mendelian Randomization website found here (https://yanglab.westlake.edu.cn/software/smr/#DataResource). The Summary statistics mendelian randomization data generated in this study are provided in the Supplementary Data 11a-c. (5) eQTL annotations used in the Transcriptome Wide Analysis as part of the current study is available at https://github.com/hakyimlab/MetaXcan. Results of the TWAS data generated in this study are provided in the Supplementary Data 12a-m. (6) eQTL annotations for FOCUS transcriptome-wide fine-mapping analysis used in the study is available at https://github.com/bogdanlab/focus. The FOCUS fine-mapping data generated in this study are provided in the Supplementary Data 13a, b. (7) Gene Ontologies within the Molecular Signature Database 7.2 used in this study are available at https://www.gsea-msigdb.org/gsea/msigdb. Gene-set analysis results based on the Molecular Signature Database are provided in the Supplementary Data 16a. (9) DrugBank annotations for WebGestalt analysis is available at. https://go.drugbank.com. Webgestalt analysis results based on the Drugbank annotations are provided in Supplementary Data 16b. (10) Brainspan data used in this study are available at https://www.brainspan.org/static/download.html. The results generated in this study based on the BrainSpan data Supplementary Data 17. (11) Data from the Allen Brain Atlas was visualized through the BrainScope Visualizer. Image annotations reported in the current study is available at https://brainscope.lumc.nl/brainscope. Results based on Brainscope visualizer is reported in Supplementary Figs. 10–14. (12) GWAS catalog annotations for gene annotations are available at https://www.ebi.ac.uk/gwas/docs/file-downloads. Gene annotations based on GWAS catalog is reported in Supplementary Data 18. (13) All other GWAS summary statistics pertaining to psychopathology traits described in Supplementary Data 1 is available for download at https://pgc.unc.edu/for-researchers/download-results/. Individual level genotype data are restricted due to data privacy laws. Requests for de-identified genotype data should be made to respective data-access committees.

## Code availability

Wrapper for GWAS summary statistics QC is available at https://github.com/maxzylam/SumstatsQC. Wrapper for local genetic correlations is available at https://github.com/maxzylam/rho-HESS-wrapper. Other codes are available as part of their software packages indicated in the following section.
