# Supplementary material for: Collective genomic segments with differential pleiotropic patterns between cognitive dimensions and psychopathology
Source: Nat Commun. 2022 Nov 11;13:6868. doi: 10.1038/s41467-022-34418-y (PMC9652380; doi:10.1038/s41467-022-34418-y)
Supplement: Supplementary file 1 — Supplementary Information [file 41467_2022_34418_MOESM1_ESM.pdf]

Supplementary Information

Collective genomic segments with differential pleiotropic patterns between cognitive dimensions and psychopathology

Authors: Max Lam[1,2,3,4,5], Chia-Yen Chen[6], W. David Hill[7], Charley Xia[7], Ruoyu Tian[8], Daniel F. Levey[9,10], Joel Gelernter[9,10, 11, 12], Murray B. Stein[13, 14, 15], Alexander S. Hatoum[16], Hailiang Huang[3,4], Anil K. Malhotra[1,2,17, 18], Heiko Runz[6], Tian Ge[3,19, 20], Todd Lencz*[1,2,17, 18]

Affiliations:

1. Division of Psychiatry Research, The Zucker Hillside Hospital, Northwell, Glen Oaks, NY, USA
2. Institute of Behavioral Science, Feinstein Institutes for Medical Research, Manhasset, NY, USA
3. Stanley Center for Psychiatric Research, Broad Institute of MIT and Harvard, Cambridge, MA, USA
4. Analytical and Translational Genetics Unit, Massachusetts General Hospital, Boston, MA, USA
5. Institute of Mental Health, Singapore, Singapore
6. Translational Biology, Research and Development, Biogen Inc., Cambridge, MA, USA
7. Lothian Birth Cohorts group, Department of Psychology, University of Edinburgh, Edinburgh, UK
8. Computational Biology and Human Genetics, Dewpoint Therapeutics, Boston, MA, USA
9. Department of Psychiatry, Yale University School of Medicine, New Haven, CT, USA
10. VA Connecticut Healthcare System, West Haven, CT, USA
11. Department of Genetics, Yale University School of Medicine, New Haven, CT, USA
12. Department of Neuroscience, Yale University School of Medicine, New Haven, CT, USA
13. VA San Diego Healthcare System, San Diego, CA, USA
14. Department of Psychiatry, University of California, San Diego, La Jolla, CA, USA
15. Herbert Wertheim School of Public Health and Human Longevity Science, University of California San Diego, La Jolla, CA, USA
16. Department of Psychiatry, University of Washington St. Louis Medical School
17. Department of Psychiatry, Zucker School of Medicine at Hofstra/Norwell, Hempstead, NY, USA
18. Department of Molecular Medicine, Zucker School of Medicine at Hofstra/Norwell, Hempstead, NY, USA
19. Psychiatric and Neurodevelopmental Genetics Unit, Center for Genomic Medicine, Massachusetts General Hospital, Boston, MA, USA
20. Department of Psychiatry, Massachusetts General Hospital, Harvard Medical School, Boston, MA, USA

*To whom correspondence should be addressed:

Todd Lencz, Zucker Hillside Hospital, Division of Psychiatry Research, 75-59 263rd Street, Glen Oaks, NY, 11004, USA, E-mail: tlencz@northwell.edu.

Members of the Biogen Biobank Team
Research and Development, Biogen Inc., Cambridge, MA, USA

Steering team: Ellen A. Tsai, Sally John, Heiko Runz

Data management team: Eric Marshall, Mehool Patel, Saranya Duraisamy

Extended Scientific team: Denis Baird, Chia-Yen Chen, Susan Eaton, Jake Gagnon, Feng Gao, Cynthia Gubbels, Yunfeng Huang, Varant Kupelian, Stephanie Loomis, Helen McLaughlin, Adele Mitchell, Benjamin Sun

Supplementary Figures and Captions

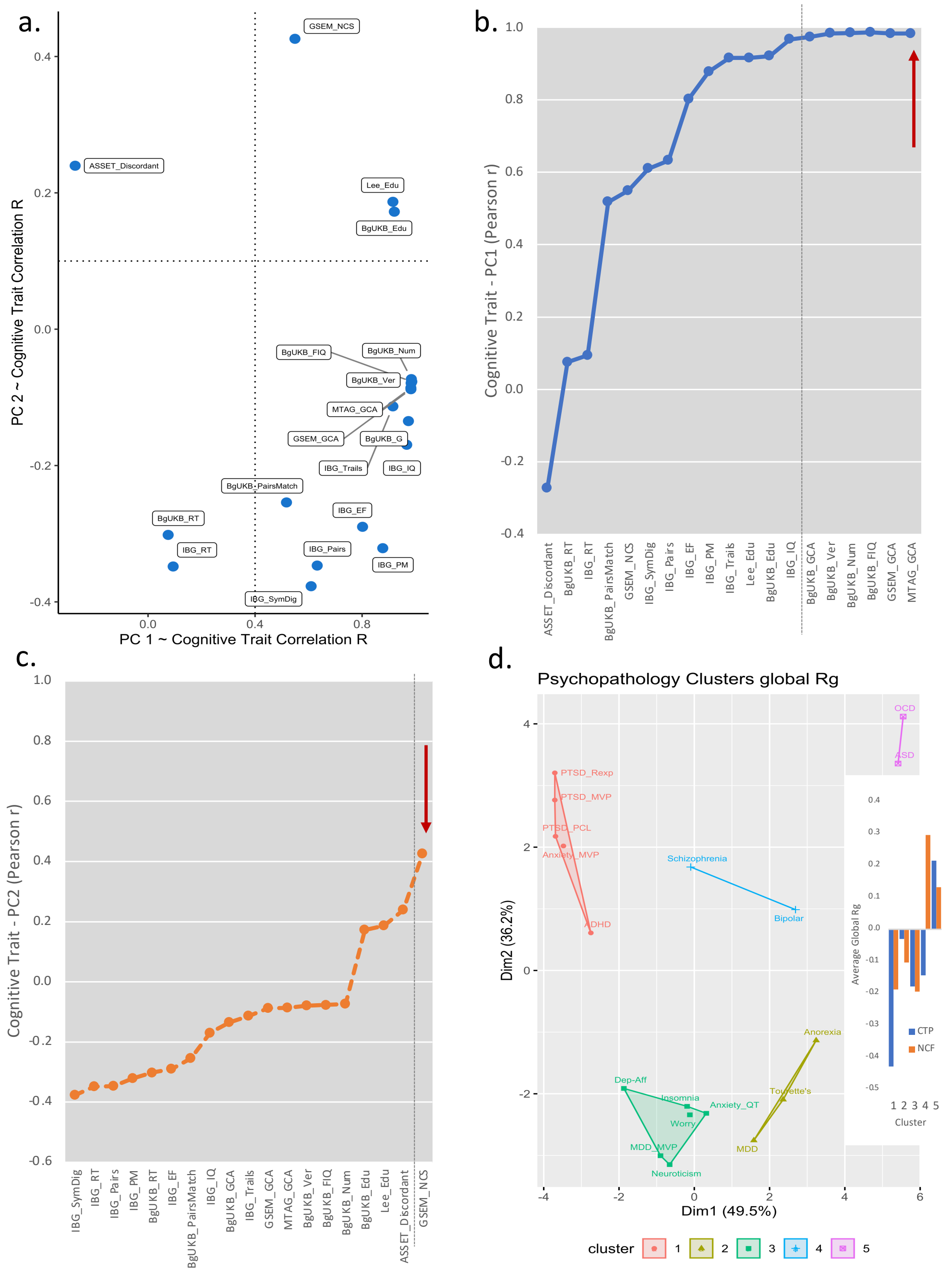

Supplementary Figure 1. Global Genetic Correlations and Dimension Reduction Procedures
Panel (a). Principal Components Analysis and loadings of cognitive traits on top two extracted PCs. Panel (b). The similarity of each cognitive trait to Cognitive Task Performance (CTP) in context of its relationship to the vector of 19 psychopathological traits. Panel (c). The similarity of each cognitive trait to NCF given its relationship to the vector of 19 psychopathological traits. Panel (d). Partitioned k-medoid cluster analysis for 18 psychopathological traits. Cluster 1: PTSD: Post-Traumatic Stress Disorder, Rexp: Re-Experiencing symptoms, MVP: Million Veteran Project, PCL: Total PCL Symptom Scores, Anxiety: Anxiety Disorder. Cluster 2: MDD: Major Depressive Disorder (Howard et al., 2019), Tourette's: Tourette's Syndrome, Anorexia: Anorexia Nervosa. Cluster 3: Dep-Aff: Depressive Affect, Anxiety_QT: Anxiety Disorder Symptom Factor Scores, MDD_MVP: Major Depressive Disorder (Million Veteran Project). Cluster 4: Bipolar: Bipolar Disorder.

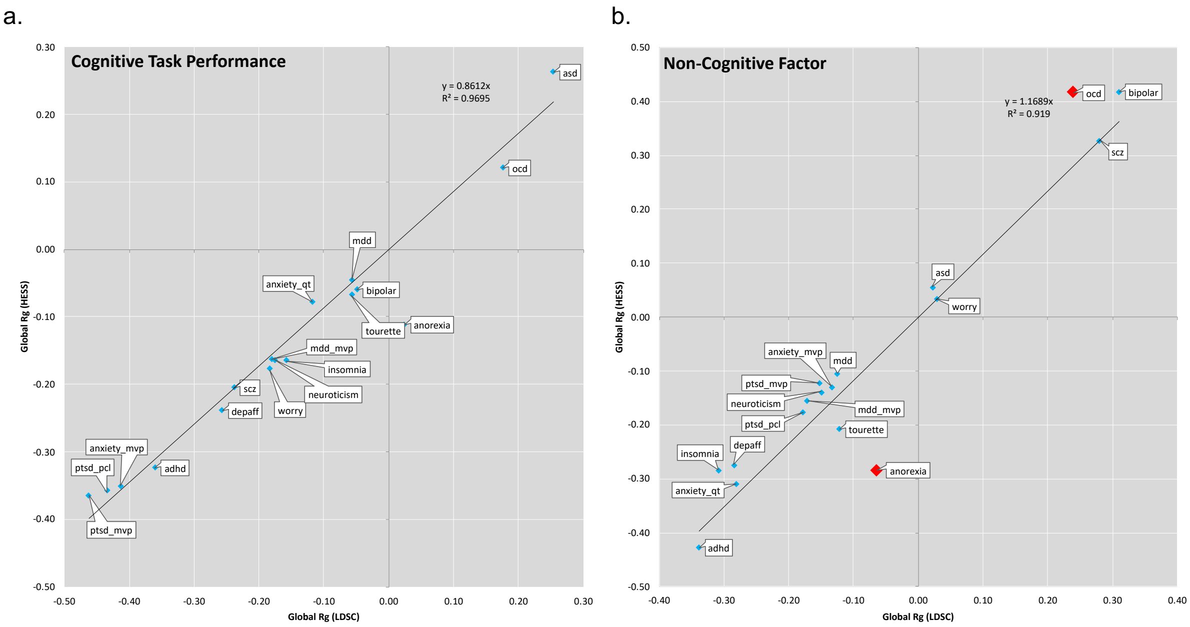

Supplementary Figure 2. Comparing global genetic correlations

Panels (a) and (b). x-axis genetic correlations estimated by LDSC; y-axis genetic correlations estimated by ρ-HESS . Intercept was set to 0 for both reference lines. anxiety_qt: Anxiety Symptom Factor Scores, anxiety_MVP: Anxiety Disorder from Million Veteran Project, asd: Autism Spectrum Disorder, ptsd_pcl: Post-Traumatic Stress Disorder - Total PCL scores, ptsd_mvp: Post-Traumatic Stress Disorder case control, both PTSD phenotypes were from the Million Veteran Project, adhd: Attention Deficit/Hyperactivity Disorder, mdd_mvp: Major Depressive Disorder from the Million Veteran Project, depaff: Depressive Affect, mdd: Major Depressive Disorder (Howard et al., 2019), scz: Schizophrenia (PGC3), tourette : Tourette's Syndrome, anorexia: Anorexia Nervosa, bipolar: Bipolar Disorder. Panel (b). Red highlighted points were anorexia (Anorexia Nervosa) and OCD.

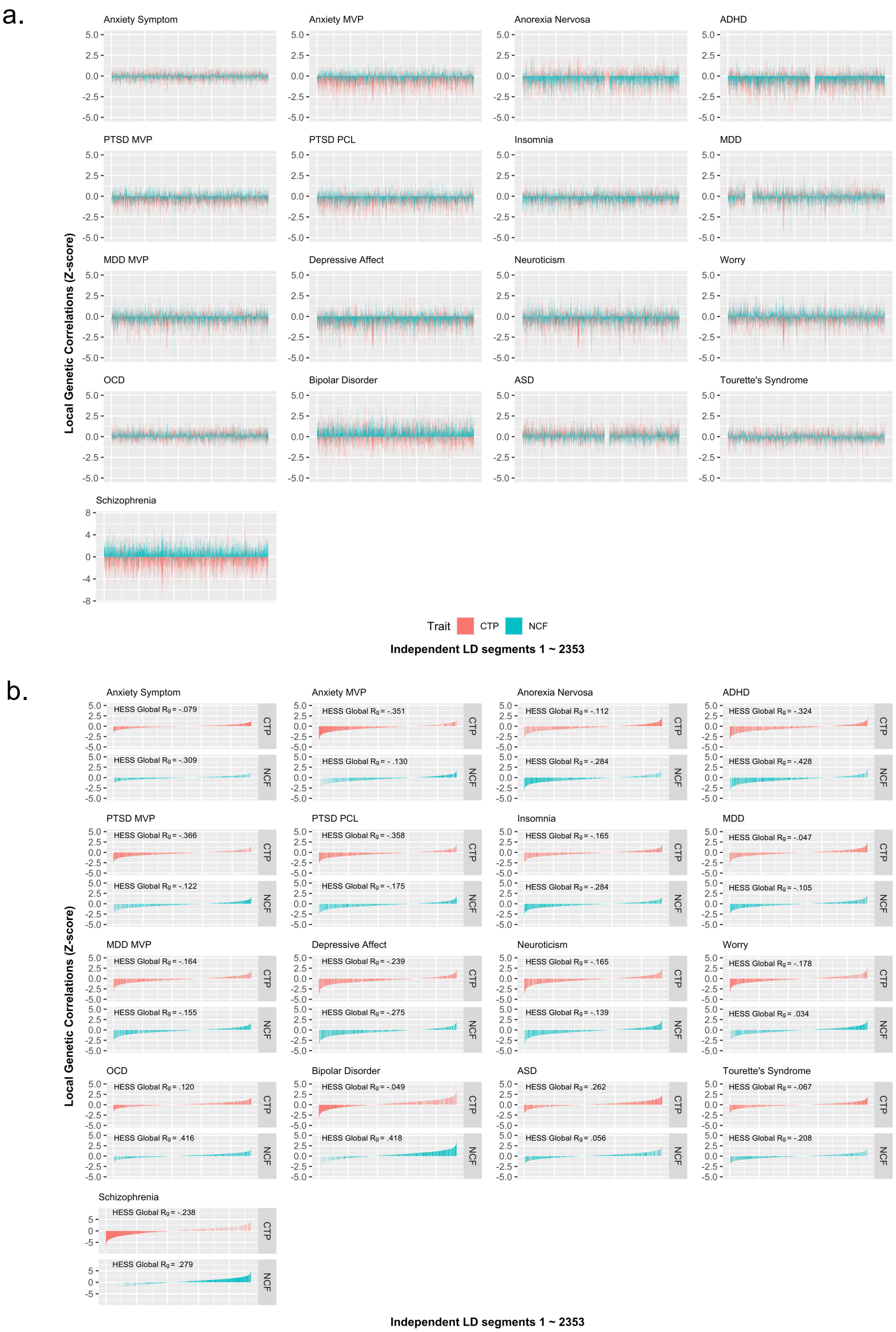

Supplementary Figure 3. Manhattan plots for ρ-HESS local genetic correlation output

Panel (a). Independent LD segments were aligned based on their chromosome and e base-pair positions sequentially. Panel (b). Local genetic correlation results and LD independent segments were aligned based on the lowest Z score to highest Z score. Panels (a) and (b): CTP – Cognitive Task Performance (in red) and NCF – Non-Cognitive Factor (in turquoise). The y-axis of all traits except for schizophrenia was set to Z score between -5 to 5. Plots for schizophrenia have y-axis limits between -7.5 and 7.5. Anxiety Symptom: Anxiety Symptom Factor Scores, Anxiety MVP: Anxiety Disorder from Million Veteran Project, MDD_MVP: Major Depressive Disorder from the Million Veteran Project, MDD: Major Depressive Disorder (Howard et al., 2019),

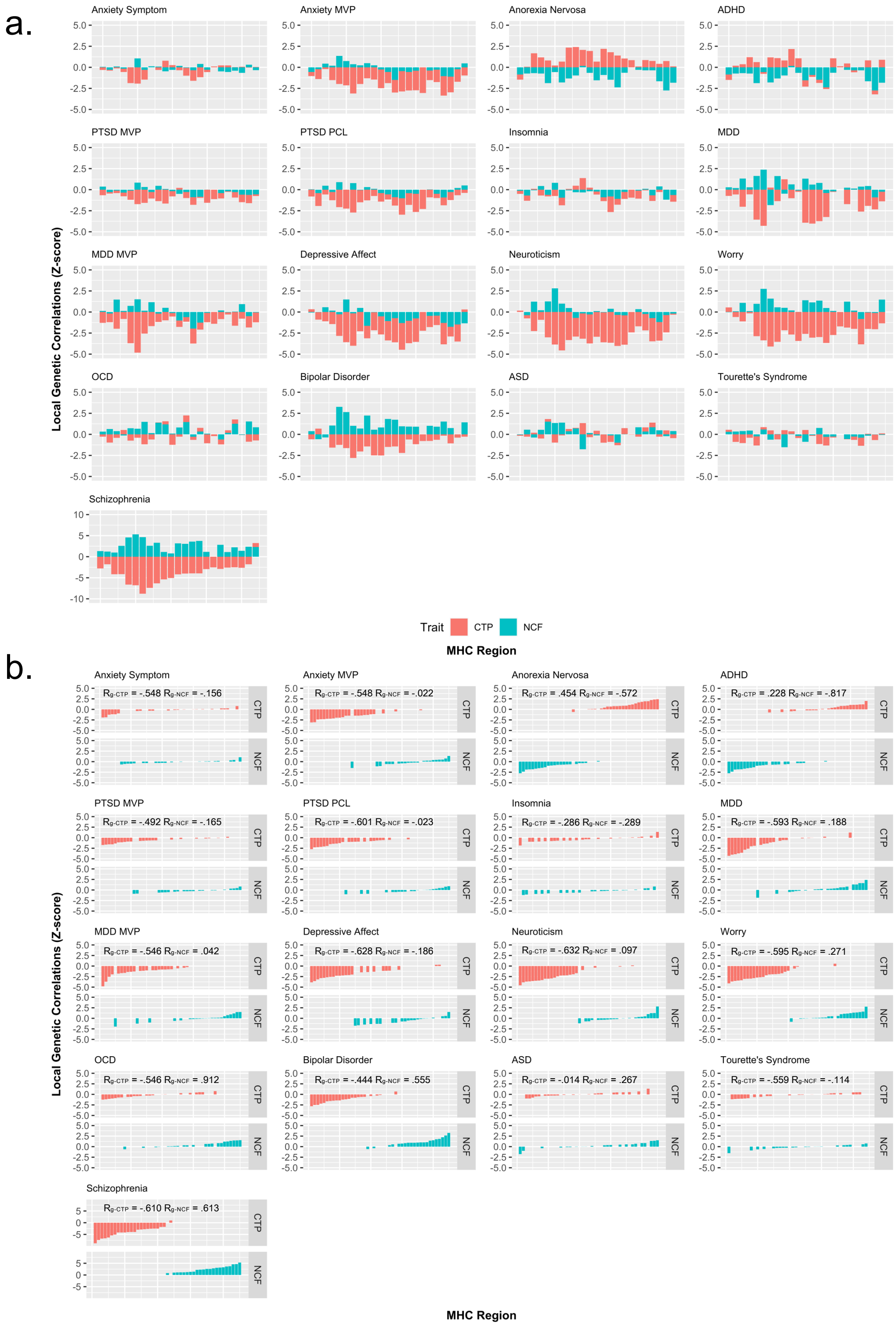

Supplementary Figure 4. Manhattan plots for ρ-HESS local genetic correlation output – MHC region
Panel (a). Independent LD segments were aligned based on their chromosome and base-pair positions sequentially. Panel (b). Local genetic correlation results and LD independent segments were aligned based on the lowest Z score to highest Z score. Panels (a) and (b): CTP – Cognitive Task Performance (in red) and NCF – Non-Cognitive Factor (in turquoise). The y-axis of all traits except for schizophrenia was set to Z score between -5 to 5. Plots for schizophrenia have y-axis limits between -10 and 10. Anxiety Symptom: Anxiety Symptom Factor Scores, Anxiety MVP: Anxiety Disorder from Million Veteran Project, MDD_MVP: Major Depressive Disorder from the Million Veteran Project, MDD: Major Depressive Disorder (Howard et al., 2019),

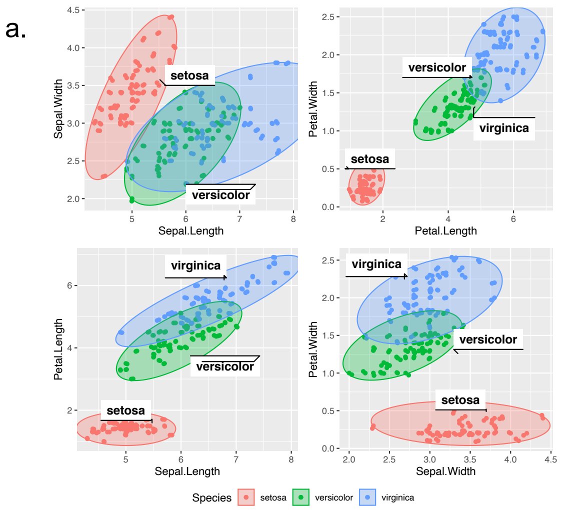

b.

UMAP models | Silhouette Scores	5 model solution	10 model solution	20 model solution
Mean	0.28	0.08	0.12
Median	0.66	0.53	0.51
75th Percentile	0.88	0.88	0.81
Maximum	0.99	0.99	0.99

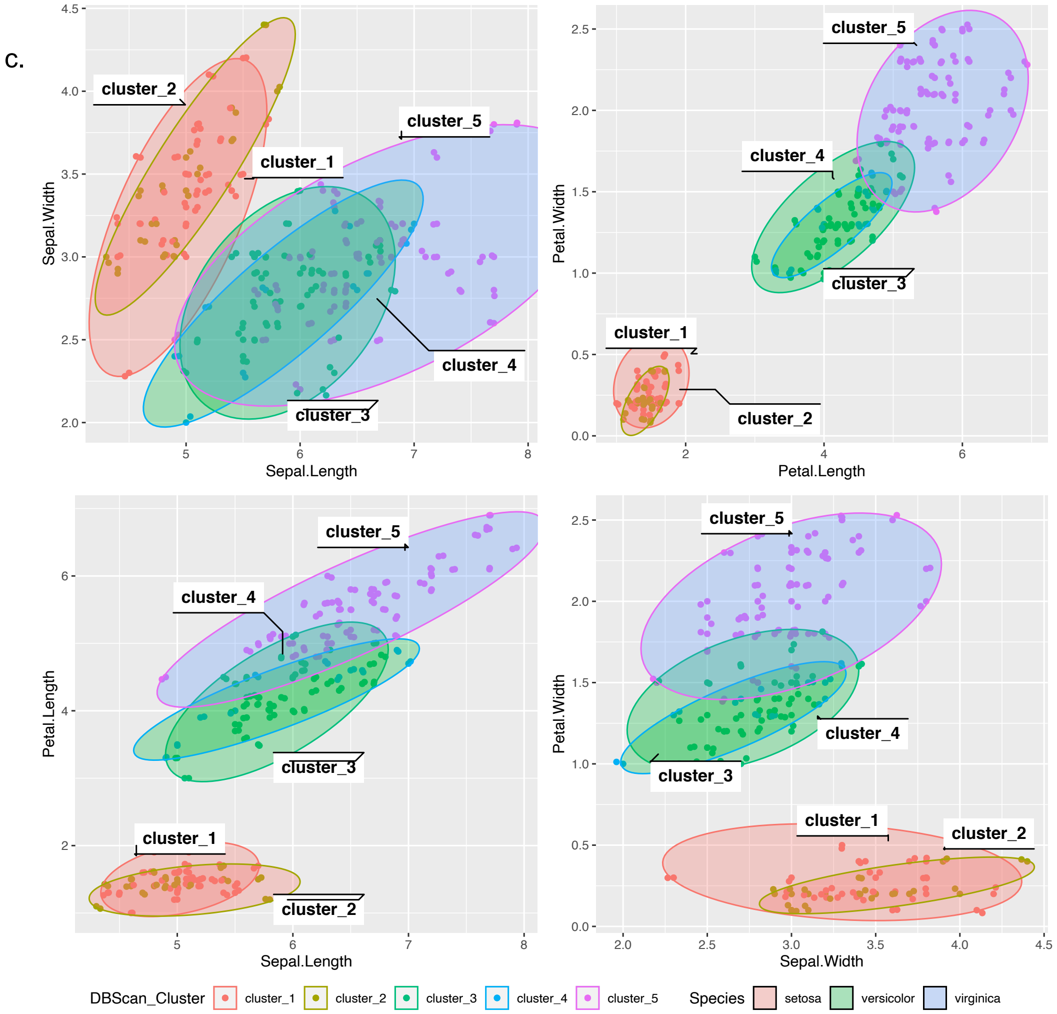

Supplementary Figure 5. DBSCAN approaches for Iris Dataset.
(a) Scatterplots for Sepal, Petal, Length and Width features clustered by ground truth categories. (b) Table of DBSCAN fit metrices – 5 model solution with 10 input features generated the best DBSCAN clustering. (c) Scatterplots for DBSCAN clusters by clustering features.

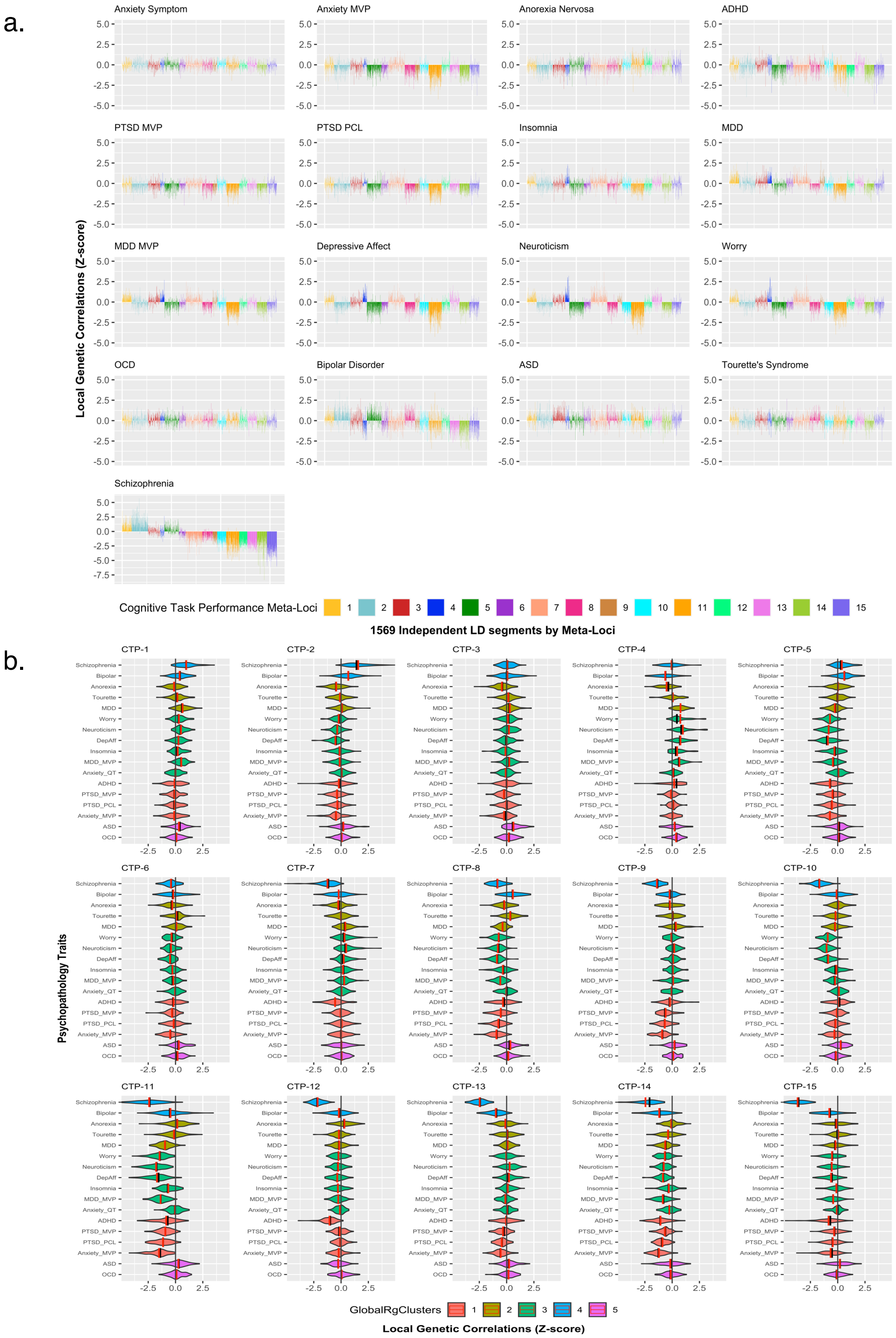

Supplementary Figure 6. Distributional patterns for local genetic correlation stratified by meta-loci for Cognitive Task Performance
(a) Meta-loci stratification by genomic coordinates between Cognitive Task Performance and each psychopathological trait. Bipolar: Bipolar Disorder, Anorexia: Anorexia Nervosa, Tourette's: Tourette's Syndrome, MDD: Major Depressive Disorder (Howard et al., 2019), Dep-Aff: Depressive Affect, MDD_MVP: Major Depressive Disorder (Million Veteran Project), Anxiety_QT: Anxiety Disorder (Symptom Factor Scores), PTSD_MVP: Post-Traumatic Stress Disorder (Million Veteran Project), PTSD_PCL: Post-Traumatic Stress Disoder (Million Veteran Project, Total PCL symptom scores), Anxiety_MVP: Anxiety Disorder (Million Veteran Project), ASD: Autism Spectrum Disorder, OCD: Obsessive Compulsive Disorder. (b) Local genetic correlation distribution for Cognitive Task Performance within each meta-locus.

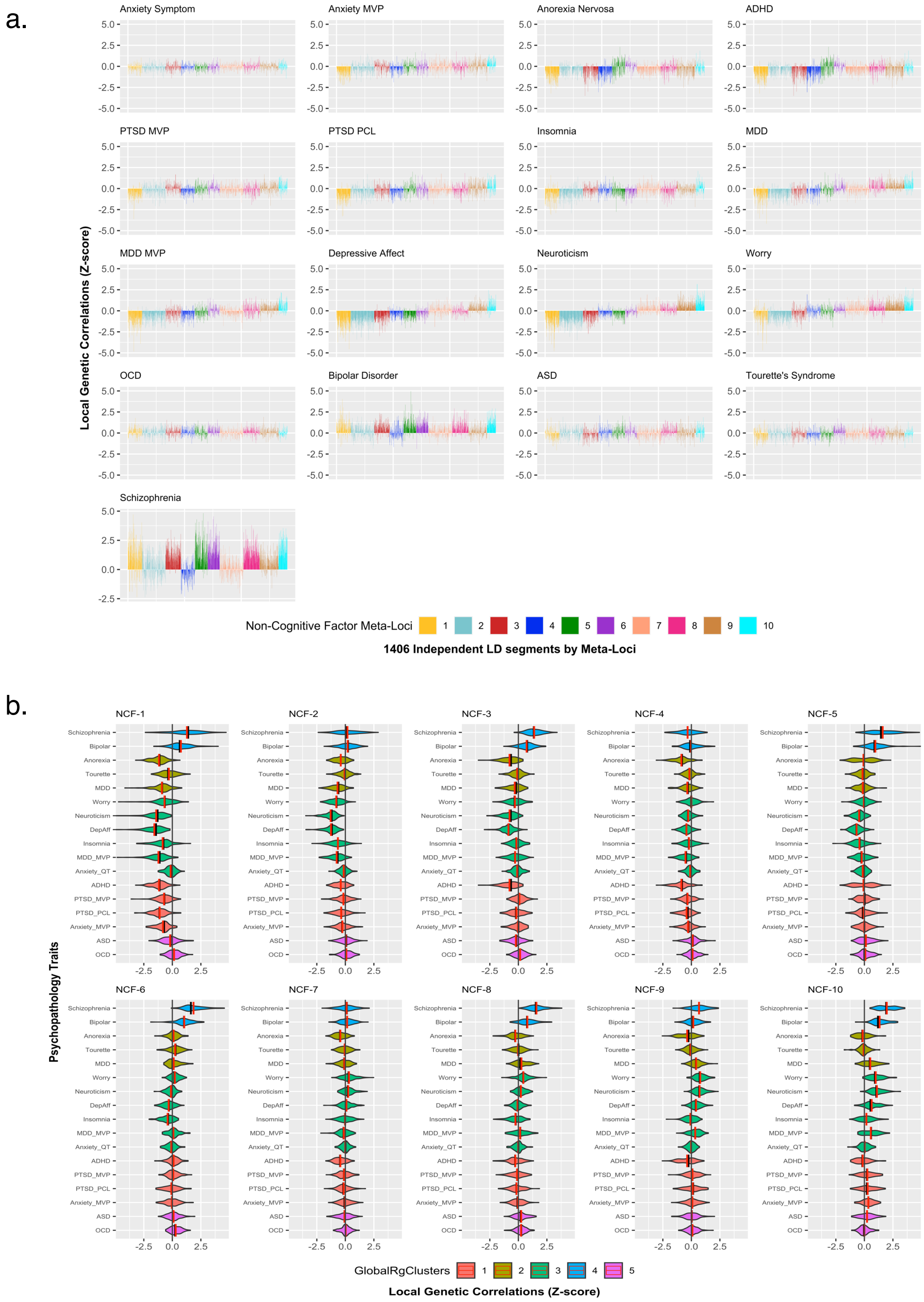

Supplementary Figure 7. Distributional patterns for local genetic correlation stratified by meta-loci for the Non-Cognitive Factor (a) Meta-loci stratification by genomic coordinates between non-cognitive factor and each psychopathological trait. Bipolar: Bipolar Disorder, Anorexia: Anorexia Nervosa, Tourette's: Tourette's Syndrome, MDD: Major Depressive Disorder (Howard et al., 2019), Dep-Aff: Depressive Affect, MDD_MVP: Major Depressive Disorder (Million Veteran Project), Anxiety_QT: Anxiety Disorder (Symptom Factor Scores), PTSD_MVP: Post-Traumatic Stress Disorder (Million Veteran Project), PTSD_PCL: Post-Traumatic Stress Disoder (Million Veteran Project, Total PCL symptom scores), Anxiety_MVP: Anxiety Disorder (Million Veteran Project), ASD: Autism Spectrum Disorder, OCD: Obsessive Compulsive Disorder. (b) Local genetic correlation distribution for non-cognitive factor within each meta-locus.

a.

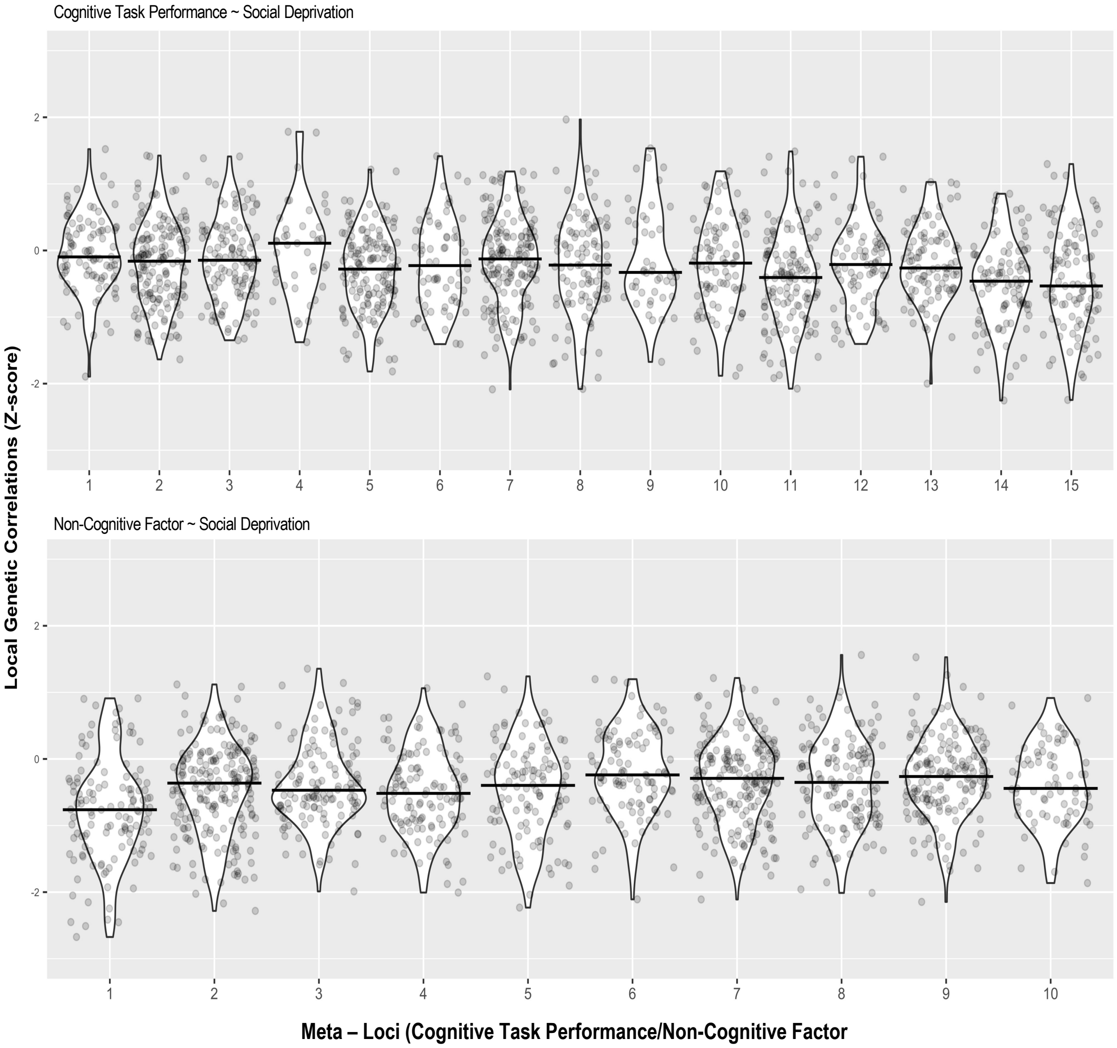

b.

CTP Meta-Locus comparison		p.adj	p.adj.signif	NCF Meta-Locus comparison		p.adj	p.adj.signif
1	11	0.0059	**	1	2	0.000629	***
1	14	0.000143	***	1	3	4.13E-05	****
1	15	0.00274	**	1	4	0.0335	*
10	14	0.0464	*	1	5	0.0136	*
11	4	0.0128	*	1	6	1.23E-09	****
14	2	0.0185	*	1	7	5.88E-08	****
14	3	0.00322	**	1	8	1.30E-05	****
14	4	0.000642	***	1	9	4.08E-09	****
14	7	0.0014	**	1	10	0.00348	**
15	3	0.0485	*	4	6	0.0334	*
15	4	0.00586	**				
15	7	0.0281	*				

Supplementary Figure 8. Distribution of local genetic correlation by LD segments between CTP/NCF and Social Deprivation
(a) Upper panel: distributional patterns of local genetic correlation with social deprivation and cognitive task performance; Lower panel: distribution patterns of local genetic correlation with social deprivation and non-cognitive factor. All post-hoc comparisons were Bonferroni adjusted (b) post-hoc pairwise t-test comparisons for meta-loci for CTP and NCF. All p-values are two-sided unless otherwise stated.

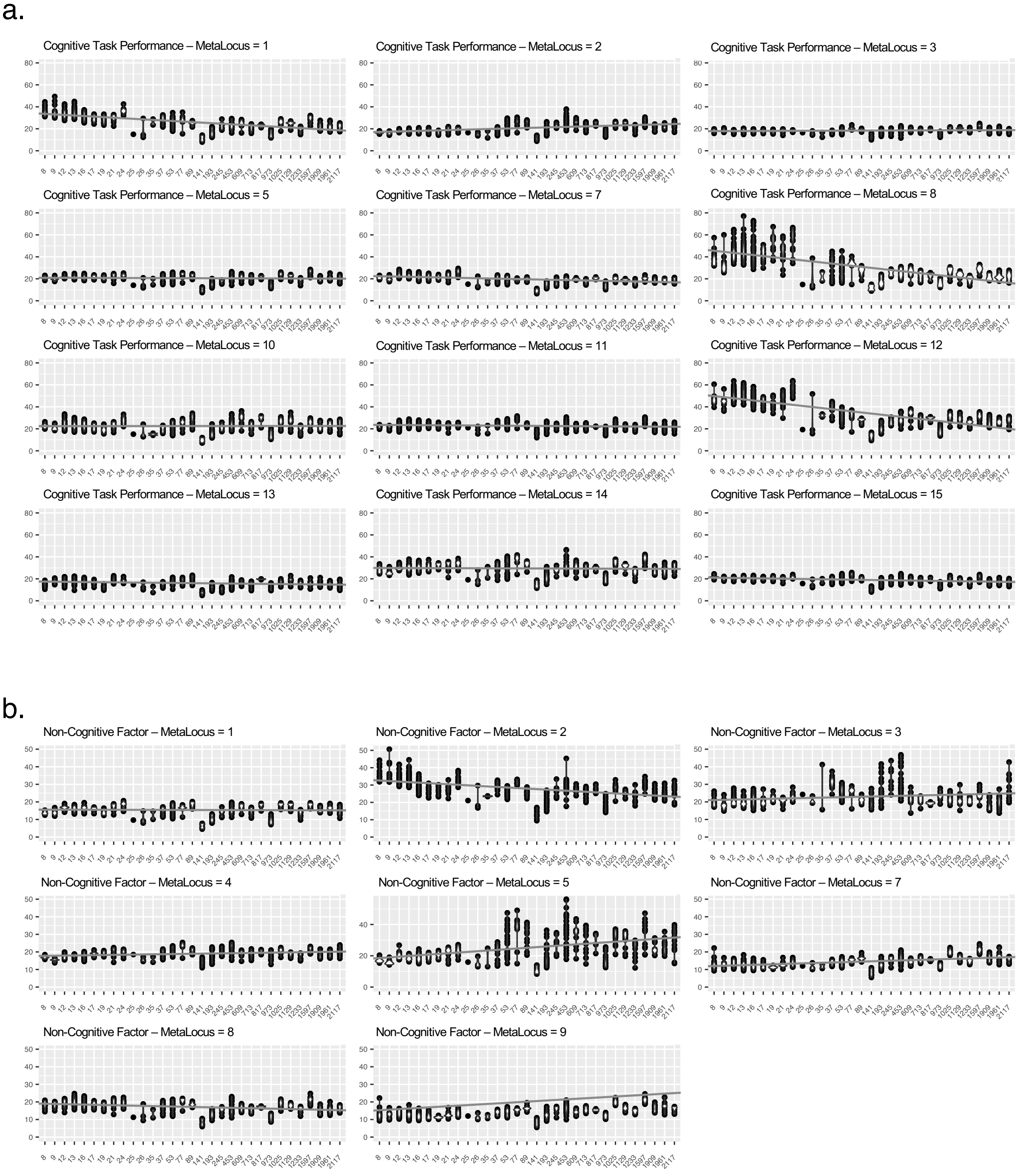

Supplementary Figure 9. Temporal Brain Expression per Meta-locus for Cognitive Task Performance and Non-Cognitive Factor
(a) temporal brain expression trajectories for Cognitive Task Performance (b) temporal brain expression trajectories for Non-Cognitive Factor. For each plot in panels (a) and (b) y-axis represents normalized aggregated gene expression values and x-axis represents time converted to weeks – ranging from 8 prenatally to 2117 weeks in adulthood.

Axon Development	BrainScope Plot1	BrainScope Plot2	BrainScope Plot3
CTP1	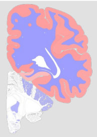	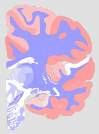	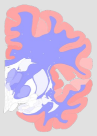
CTP2	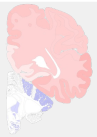	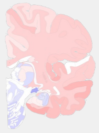	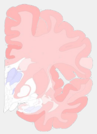
CTP5	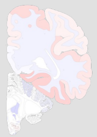	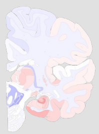	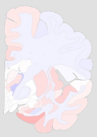
CTP11	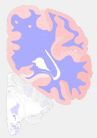	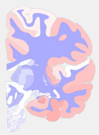	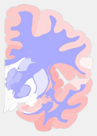
CTP12	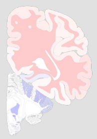	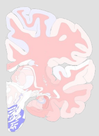	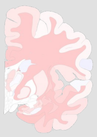
CTP13	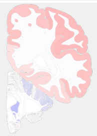	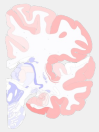	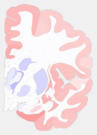
CTP15	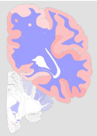	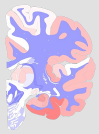	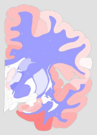

Supplementary Figure 10. Spatial distribution of gene expression across meta-loci from Allen Brain Atlas for Axon Development.
Red color within the brain slices suggest up-regulated gene expression in the brain, and blue represents areas with down-regulated gene expression.

Cell Morphogenesis involved in Neuron Differentiation	BrainScope Plot1	BrainScope Plot2	BrainScope Plot3
CTP1	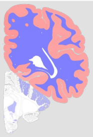	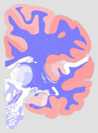	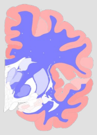
CTP2	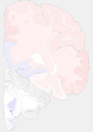	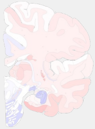	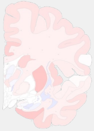
CTP5	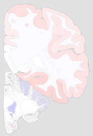	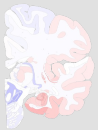	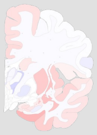
CTP11	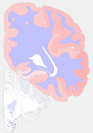	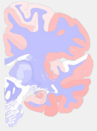	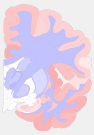
CTP12	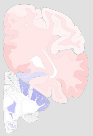	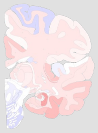	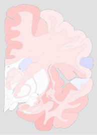
CTP13	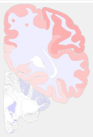	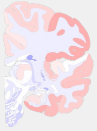	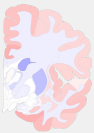
CTP14	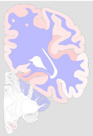	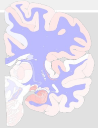	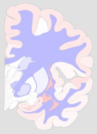
CTP15	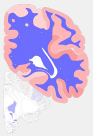	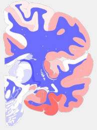	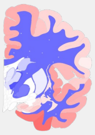

Supplementary Figure 11. Spatial distribution of gene expression across meta-loci from Allen Brain Atlas for Cell Morphogenesis involved in Neuron Differentiation.
Red color within the brain slices suggest up-regulated gene expression in the brain, and blue represents areas with down-regulated gene expression.

Neurogenesis	BrainScope Plot1	BrainScope Plot2	BrainScope Plot3
CTP1	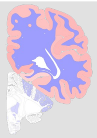	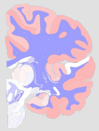	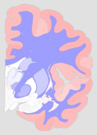
CTP2	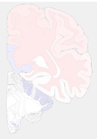	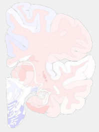	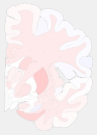
CTP5	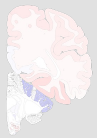	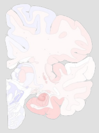	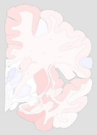
CTP7	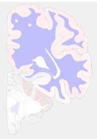	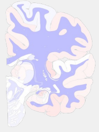	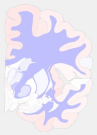
CTP11	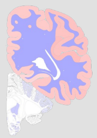	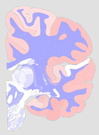	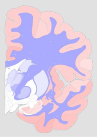
CTP12	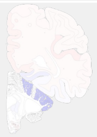	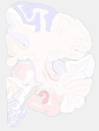	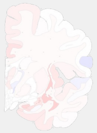
CTP15	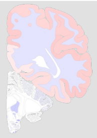	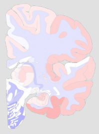	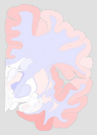

Supplementary Figure 12. Spatial distribution of gene expression across meta-loci from Allen Brain Atlas for Neurogenesis.
Red color within the brain slices suggest up-regulated gene expression in the brain, and blue represents areas with down-regulated gene expression.

Neuron Development	BrainScope Plot1	BrainScope Plot2	BrainScope Plot3
CTP2	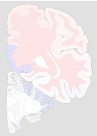	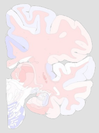	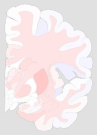
CTP5	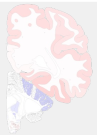	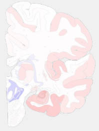	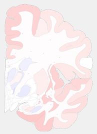
CTP7	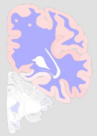	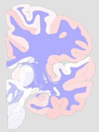	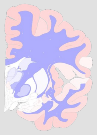
CTP11	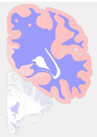	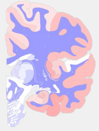	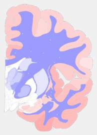
CTP12	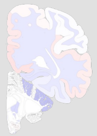	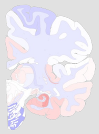	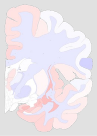
CTP13	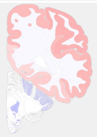	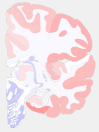	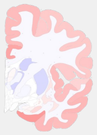
CTP15	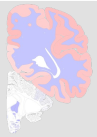	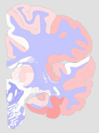	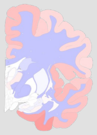

Supplementary Figure 13. Spatial distribution of gene expression across meta-loci from Allen Brain Atlas for Neurogenesis.
Red color within the brain slices suggest up-regulated gene expression in the brain, and blue represents areas with down-regulated gene expression.

Neuron Differentiation	BrainScope Plot1	BrainScope Plot2	BrainScope Plot3
CTP1	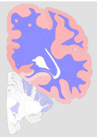	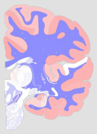	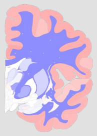
CTP2	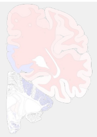	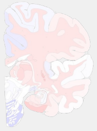	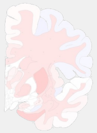
CTP5	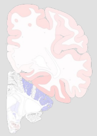	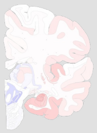	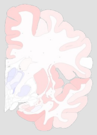
CTP7	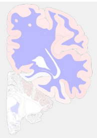	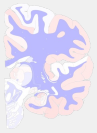	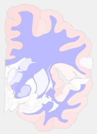
CTP11	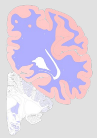	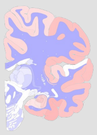	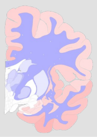
CTP12	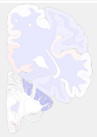	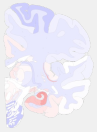	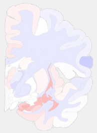
CTP13	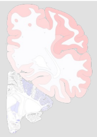	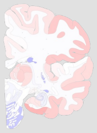	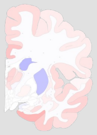
CTP15	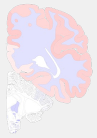	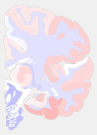	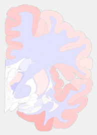

Supplementary Figure 14. Spatial distribution of gene expression across meta-loci from Allen Brain Atlas for Neuron Differentiation.
Red color within the brain slices suggest up-regulated gene expression in the brain, and blue represents areas with down-regulated gene expression.
