# Supplementary material for: Collective genomic segments with differential pleiotropic patterns between cognitive dimensions and psychopathology
Source: Nat Commun. 2022 Nov 11;13:6868. doi: 10.1038/s41467-022-34418-y (PMC9652380; doi:10.1038/s41467-022-34418-y)
Supplement: Supplementary file 2 — Description of Additional Supplementary Files [file 41467_2022_34418_MOESM2_ESM.pdf]

Description of Additional Supplementary Files

File Name: Supplementary Data 1
Description: Description of GWAS Summary Statistics

File Name: Supplementary Data 2
Description: **a**. Global Genetic Correlations of Cognitive Traits and Psychopathology
Bg : Biogen Inc., these are closed access summary statistics from the UK Biobank obtained via collaboration with Biogen Pharmaceuticals; GSEM: GenomicSEM, these are original summary statistics obtained from the Demange et al., 2021 Nature Genetics report. IBG: Institute of Behavioral Genetics, these are GWAS summary statistics of phenotypically imputed cognitive data based on the UK Biobank obtained from Hatoum et al., 2020, reported in MedRxiv. ASSET: These are GWAS summary statistics emanating from ASSET analysis reported in Lam et al., 2019, reported in American Journal of Human Genetics. Acronyms for cognitive traits: Edu : Education Attainment, FIQ: Fluid IQ, Num: Numerical reasoning, Ver: Verbal reasoning, GCA: General cognitive ability (Spearman's G), EF: Executive function, Pairs: Pairs matching PM: Prospective memory, RT: Reaction time, SymDig: Symbol Digit, Trails: Trail Making Test; Dim1 and 2: these are PCA dimensions derived from the correlation matrix (See also TableS2b); cluster: K-mediod partitioning clusters (See also Figure 2, and TabeS3); Dim1 & 2 ~ Cognitive Trait: Pearson correlations loadings of cogniive trait on PC Dimensions 1 and 2. min & max Rg: Minimum and maximum Rg for each cognitive-psychopathological trait pair

b. K-Mediod Clustering and PCA Dimensions 1 and 2
Bg : Biogen Inc., these are closed access summary statistics from the UK Biobank obtained via collaboration with Biogen Pharmaceuticals; GSEM: GenomicSEM, these are original summary statistics obtained from the Demange et al., 2021 Nature Genetics report. IBG: Institute of Behavioral Genetics, these are GWAS summary statistics of phenotypically imputed cognitive data based on the UK Biobank obtained from Hatoum et al., 2020, reported in MedRxiv. ASSET: These are GWAS summary statistics emanating from ASSET analysis reported in Lam et al., 2019, reported in American Journal of Human Genetics. Acronyms for cognitive traits: Edu : Education Attainment, FIQ: Fluid IQ, Num: Numerical reasoning, Ver: Verbal reasoning, GCA: General cognitive ability (Spearman's G), EF: Executive function, Pairs: Pairs matching PM: Prospective memory, RT: Reaction time, SymDig: Symbol Digit, Trails: Trail Making Test; Dim1 and 2: these are PCA dimensions derived from the correlation matrix (See also TableS2b); cluster: K-mediod partitioning clusters (See also Figure 2, and TabeS3); Dim1 & 2 ~ Cognitive Trait: Pearson correlations loadings of cogniive trait on PC Dimensions 1 and 2. min & max Rg: Minimum and maximum Rg for each cognitive-psychopathological trait pair

c. Fit Statistics for Global R_G clustering

File Name: Supplementary Data 3
Description: Local heritability of Cognitive Task Performance and Non-Cognitive Factor. The total heritability based on the sum of all 2353 independent LD segments is h^2 = 0.23 for Cognition and h^2 = 0.31 for Non-Cognitive Factor; num_snp = number of snps use for estimation of local genetic correlations; k = number of eigen vectors for single value

decomposition; local_h2g = local heritability as estimated via HESS; var = estimated variance; p = two-sided p-values

File Name: Supplementary Data 4
Description: Local genetic correlations estimated by HESS for 17 Cognitive Task Performance - Psychopathology trait pairs.
Heritability indicated for each region was for Cognitive Task Performance - the strength of the heritability was colored in indigo. For each of the columns representing z-scores, each cell was colored based on their direction. Positive Z-scores were colored red, and negative Z-scores were colored blue. Columns were defined as follows, Anxiety_QT: Anxiety Symptoms Factor Scores, Anxiety_MVP: Anxiety Disorder from Million Veteran Project, PTSD_PCL: Post-Traumatic Stress Disorder - Total PCL scores, PTSD_MVP: Post-Traumatic Stress Disorder case control, both PTSD phenotypes were from the Million Veteran Project, ADHD: Attention Deficit/Hyperactivity Disorder, MDD_MVP: Major Depressive Disorder from the Million Veteran Project, Dep-Aff: Depressive Affect, MDD: Major Depressive Disorder (Howard et al., 2019), Tourette's : Tourette's Disorder, Anorexia: Anorexia Nervosa, Bipolar Disorder. Each phenotype column was color coded based on K-medoid clustering for global genetic correlations. V1 to V10 are UMAP dimensions. DB cluster are clusters extracted from local genetic correlations, while H-cluster are meta-loci derived from hierarchical clustering of local genetic correlations, DB cluster and UMAP dimensions to recover global clusters of LD independent regions.

File Name: Supplementary Data 5
Description: Local genetic correlations estimated by HESS for 17 Non-Cognitive Factor - Psychopathology trait pairs.
Heritability indicated for each region was for Cognitive Task Performance - the strength of the heritability was colored in indigo. For each of the columns representing z-scores, each cell was colored based on their direction. Positive Z-scores were colored red, and negative Z-scores were colored blue. Columns were defined as follows, Anxiety_QT: Anxiety Symptoms Factor Scores, Anxiety_MVP: Anxiety Disorder from Million Veteran Project, PTSD_PCL: Post-Traumatic Stress Disorder - Total PCL scores, PTSD_MVP: Post-Traumatic Stress Disorder case control, both PTSD phenotypes were from the Million Veteran Project, ADHD: Attention Deficit/Hyperactivity Disorder, MDD_MVP: Major Depressive Disorder from the Million Veteran Project, Dep-Aff: Depressive Affect, MDD: Major Depressive Disorder (Howard et al., 2019), Tourette's : Tourette's Disorder, Anorexia: Anorexia Nervosa, Bipolar Disorder. Each phenotype column was color coded based on K-medoid clustering for global genetic correlations. V1 to V10 are UMAP dimensions. DB cluster are clusters extracted from local genetic correlations, while H-cluster are meta-loci derived from hierarchical clustering of local genetic correlations, DB cluster and UMAP dimensions to recover global clusters of LD independent regions.

File Name: Supplementary Data 6
Description: Local Genetic Correlations for Cognitive Task Performance/Non-Cognitive Factor and Psychopathological traits with effect sizes |Z| > 4
Trait1: Cognitive dimensions, either Cognitive Task Performance or Non-Cognitive Factor, LOCI: number of loci from 1-2353, h^2: heritability of the cognitive dimension indicated in the Trait 1 column.

File Name: Supplementary Data 7
Description: Local Genetic Correlations for Cognitive Task Performance and Non-Cognitive Factor with Social Deprivation

File Name: Supplementary Data 8
Description: **a**. Density Based Clustering Silhouette Scores for Cognitive Task Performance
Fit statistics for density-based clustering for local genetic correlation input.
N umap dimensions: Number of UMAP dimensions used for density-based clustering, Epsilon: Nearest neighbor threshold, based on k-nearest neighbor distance; Unclassifiable: outlier LD segments not classified by density-based clustering; N db clusters: number of density-based clusters; Median: median silhouette scores, Mean: mean silhouette scores; 75th percentile: 3rd quarter silhouette scores; Max: maximum silhouette score.

b. Hierarchical Cluster Fit Indices for Cognitive Task Performance
asw: Average Silouette Width; ch: Calinski and Harabasz index; dunn: minimum separation / maximum diameter. Dunn index; cvnnd: coefficient of variation of dissimilarities to nnkth nearest within-cluster neighbour, measuring uniformity of within-cluster densities, weighted over all clusters; correlation between distances and a 0-1-vector where 0 means same cluster, 1 means different clusters; avewithin: average distance within clusters; sindex: separation index; entropy: entropy of the distribution of cluster memberships; avstability: average clusterwise stability statistics by resampling. Indices highlighted in red = top ranked index, gold = second ranked index, orange = third ranked index.

c. Descriptive Statistics for CTP meta-locus
Overall heritability for the meta-locus, number of LD independent regions that were part of the meta-locus, cumulative length of the meta-locus and percentage of the heritability accounted for, and cumulative heritability accounted for by each meta-locus are displayed.

File Name: Supplementary Data 9
Description: **a**. Density Based Clustering Silhouette Scores for Non-Cognitive Factor
Fit statistics for density-based clustering for local genetic correlation input.
N umap dimensions: Number of UMAP dimensions used for density-based clustering, Epsilon: Nearest neighbor threshold, based on k-nearest neighbor distance; Unclassifiable: outlier LD segments not classified by density-based clustering; N db clusters: number of density-based clusters; Median: median silhouette scores, Mean: mean silhouette scores; 75th percentile: 3rd quarter silhouette scores; Max: maximum silhouette score.

b. Hierarchical Cluster Fit Indices for Non-Cognitive Factor
asw: Average Silhouette Width; ch: Calinski and Harabasz index; dunn: minimum separation / maximum diameter. Dunn index; cvnnd: coefficient of variation of dissimilarities to nnkth nearest within-cluster neighbor, measuring uniformity of within-cluster densities, weighted over all clusters; correlation between distances and a 0-1-vector where 0 means same cluster, 1 means different clusters; avewithin: average distance within clusters; sindex: separation index; entropy: entropy of the distribution of cluster memberships; avstability: average clusterwise stability statistics by resampling. Indices highlighted in red = top ranked index, gold = second ranked index, orange = third ranked index.

c. Descriptive Statistics for NCF meta-loci
Overall heritability for the meta-locus, number of LD independent regions that were part of the meta-locus, cumulative length of the meta-locus and percentage of the heritability accounted for and cumulative heritability accounted for by each meta-locus are displayed.

File Name: Supplementary Data 10
Description: MAGMA Gene Based Genome Wide Analysis and PoPs gene prioritization score.
x = all columns with x suffixes denote results for Cognitive Task Performance, and all columns with y suffixes denote results for Non-Cognitive Factor
popscore = gene prioritization score estimated by PoPs. Z scores and P values are estimated via MAGMA gene analysis. P-values are two-sided unless otherwise stated.

File Name: Supplementary Data 11
Description: **a**. Summary statistics mendelian randomization results.
all columns with x suffixes denote results for Cognitive Task Performance and all columns with y suffixes denote results for Non-Cognitive Factor
Annotation: Brain-emeta

b. Summary statistics mendelian randomization results.
all columns with x suffixes denote results for Cognitive Task Performance and all columns with y suffixes denote results for Non-Cognitive Factor
Annotation: PsychENCODE HCP

c. Summary statistics mendelian randomization results.
all columns with x suffixes denote results for Cognitive Task Performance and all columns with y suffixes denote results for Non-Cognitive Factor
Annotation: PsychENCODE PEER

File Name: Supplementary Data 12
Description: Results of S-PrediXcan transcriptomic wide analysis based on GTEx8 brain tissues
all columns with x suffixes denote results for Cognitive Task Performance and all columns with y suffixes denote results for Non-Cognitive Factor
pred_perf_r2: (cross-validated) R2 of tissue model's correlation to gene's measured transcriptome (prediction performance); pred_perf_pval: pval of tissue model's correlation to gene's measured transcriptome (prediction performance); n_snps_used: number of snps from GWAS that got used in S-PrediXcan analysis; n_snps_in_cov: number of snps in the covariance matrix; n_snps_in_model: number of snps in the model; var_g: variance of the gene expression, calculated as W' * G * W (where W is the vector of SNP weights in a gene's model, W' is its transpose, and G is the covariance matrix). P-values are two-sided unless otherwise stated.
(a) Anterior Cingulate Cortex
(b) Amygdala
(c) Caudate – Basal Ganglia
(d) Cerebellum
(e) Cerebellar Hemisphere
(f) Cortex

(g) Frontal Cortex
(h) Hippocampus
(i) Hypothalamus
(j) Nucleus Accumbens
(k) Putamen – Basal Ganglia
(l) Spinal Cord
(m) Substantia Nigra

File Name: Supplementary Data 13
Description: **a**. FOCUS transcriptomic wide fine-mapping procedures
Results displayed for all Cognitive Task Performance TWAS brain tissue from focusdb
cv.R2 = Cross-validation predictive Rsquared; cv.R2.pval = P-value of the Cross-validation; twas_z = Marginal TWAS Z score; twas_z = Marginal TWAS Z score; in_cred_set = Flag indicating whether or not model is included in the credible set; region = Identifier for the genomic region; ldsegmnt = independent LD segment. P-values are two-sided unless stated.

b. FOCUS transcriptomic wide fine-mapping procedures
Results displayed for all Non-Cognitive Factor TWAS brain tissue from focusdb
cv.R2 = Cross-validation predictive Rsquared; cv.R2.pval = P-value of the Cross-validation; twas_z = Marginal TWAS Z score; twas_z = Marginal TWAS Z score; in_cred_set = Flag indicating whether or not model is included in the credible set; region = Identifier for the genomic region; ldsegmnt = independent LD segment. P-values are two-sided unless stated.

File Name: Supplementary Data 14
Description: **a**. Gene ranks and corresponding percentile
all columns with suffixes "x" denote Cognitive Task Performance, all columns with suffixes "y" denote Non-Cognitive Factor

b. Gene ranks based on multiple transcriptomic wide analysis approaches on Cognitive Task Performance and Non-Cognitive factor GWAS Summary Statistics
all columns with suffixes "x" denote Cognitive Task Performance, all columns with suffixes "y" denote Non-Cognitive Factor. Genomic coordinates of each independent LD segments are provide as CHR: chromosome, START: Start BP coordinates, END: End BP coordinates, KBSpan: Length of LD segment; ensgene: ensemble gene ID; gene: standard gene symbol; CTP.meta.locus.ID: meta-locus ID for Cognitive Task Performance; NCF.meta.locus.ID: meta-locus ID for noncognitive factor; MAGMA.rank: MAGMA gene ranking; PoPs.rank: PoPs gene ranking; TWAS.avg.gene.rank: Average gene rank across TWAS methodologies and tissue eQTL; focus.all: credible genes identified by FOCUS finemapping

File Name: Supplementary Data 15
Description: Computation of inverse rank scores based on PoPs gene scores and 50th percentile selected TWAS genes
Note:. CTP.meta.locus.ID: ID for CTP meta-locus; NCF.meta.locus.ID: ID for NCF meta-locus; PoPscore: gene prioritization score from PoPs algorithm; PoP.rank: ranking of gene from PoPscore; PoP.rank.norm; normalized rank score 0-1.

File Name: Supplementary Data 16
Description: **a.** Consolidated results of gene set analysis in long format
SIZE: final gene set size; NES: normalized enrichment score, NOM p-value: nominal p-value, FDR q-value: False discovery rate, estimated probability that the normalized enrichment score represents a false positive finding; LEADING EDGE: Tags. The percentage of gene hits before the peak in the running enrichment score. This gives an indication of the percentage of genes contributing to the enrichment score, List. The percentage of genes in the ranked gene list before the peak in the running enrichment score. This gives an indication of where in the list the enrichment score is attained, Signal. The enrichment signal strength that combines the two previous statistics. If the gene set is entirely within the first Nth positions in the list, then the signal strength is maximal or 100%. If the gene set is spread throughout the list, then the signal strength decreases towards 0%; Method: gene set analysis method, GSEA, WG – WebGestalt or FUMA::GENE2FUNC (GENE2FUNC); Metalocus.ID: meta-locus ID for either CTP or NCF; Phenotype: Cognitive Task Performance (CTP) or Non-Cognitive Factor (NCF); Category: GO Ontology Biological Processes, Molecular Function, or Cellular Component Gene sets; Neural: Annotation for which the gene set implicates the brain/neurological component; Multi-Method: Annotation if the gene set was indexed by multiple gene-set analysis methodology; n.locus: number of meta-loci that the gene-set was implicated in. ".5" occurs where at least one of the gene sets were implicated in multiple meta-loci, and multiple methodologies; driver.genes: these are genes that are implicated within the gene sets in questions. However, only driver genes within the GSEA methodology were considered for downstream analysis.

b. Consolidated results for Drug based gene set analysis (WebGestalt only)
SIZE: final gene set size, NES: normalized enrichment score, NOM p-value: nominal p-value, FDR q-value: False discovery rate, estimated probability that the normalized enrichment score represents a false positive finding; Phenotype: Cognitive Task Performance (CTP) or Non-Cognitive Factor (NCF); n.locus: number of meta-loci that the gene-set was implicated in. ".5" occurs where at least one of the gene sets were implicated in multiple meta-loci, and multiple methodologies; driver.genes: these are genes that are implicated within the gene sets in questions. However, only driver genes within the GSEA methodology were considered for downstream analysis.

File Name: Supplementary Data 17
Description: **a.** Linear mixed-model results comparing null and alternative models
Null model BS0: Expression ~ Weeks + Sex + (1 | donor_id); Alternative model BS1: Expression ~ Sex + Weeks * Trait + (1 | donor_id); Pr: Two-sided

b. Linear mixed-model results for trait by time interaction
Interaction model: Expression ~ Sex + Weeks * Trait + (1 | donor_id) ; Pr: Two-sided

c. Linear mixed-model results for time effects within each meta-locus for Cognitive Task Performance and Non-Cognitive Factor
CTP: Cognitive Task Performance; NCF: Non-Cognitive Factor; p: Two-sided.

File Name: Supplementary Data 18
Description: Driver Gene Annotations based on GWAS catalogue and Druggability Tiers for Gene Targets
