# Supplementary material for: Collective genomic segments with differential pleiotropic patterns between cognitive dimensions and psychopathology
Source: Nat Commun. 2022 Nov 11;13:6868. doi: 10.1038/s41467-022-34418-y (PMC9652380; doi:10.1038/s41467-022-34418-y)
Supplement: Supplementary file 21 — Reporting Summary [file 41467_2022_34418_MOESM21_ESM.pdf]

nature portfolio

Corresponding author(s): Todd Lencz

Last updated by author(s): Oct 19, 2022

Reporting Summary

Nature Portfolio wishes to improve the reproducibility of the work that we publish. This form provides structure for consistency and transparency in reporting. For further information on Nature Portfolio policies, see our Editorial Policies and the Editorial Policy Checklist.

Statistics

For all statistical analyses, confirm that the following items are present in the figure legend, table legend, main text, or Methods section.

n/a	Confirmed	
☐	☒	The exact sample size (*n*) for each experimental group/condition, given as a discrete number and unit of measurement
☒	☐	A statement on whether measurements were taken from distinct samples or whether the same sample was measured repeatedly
☐	☒	The statistical test(s) used AND whether they are one- or two-sided<br>*Only common tests should be described solely by name; describe more complex techniques in the Methods section.*
☒	☐	A description of all covariates tested
☐	☒	A description of any assumptions or corrections, such as tests of normality and adjustment for multiple comparisons
☐	☒	A full description of the statistical parameters including central tendency (e.g. means) or other basic estimates (e.g. regression coefficient) AND variation (e.g. standard deviation) or associated estimates of uncertainty (e.g. confidence intervals)
☐	☒	For null hypothesis testing, the test statistic (e.g. *F*, *t*, *r*) with confidence intervals, effect sizes, degrees of freedom and *P* value noted<br>*Give P values as exact values whenever suitable.*
☒	☐	For Bayesian analysis, information on the choice of priors and Markov chain Monte Carlo settings
☒	☐	For hierarchical and complex designs, identification of the appropriate level for tests and full reporting of outcomes
☐	☒	Estimates of effect sizes (e.g. Cohen's *d*, Pearson's *r*), indicating how they were calculated

Our web collection on statistics for biologists contains articles on many of the points above.

Software and code

Policy information about availability of computer code

Data collection	GWAS summary statistics were used for the current report. Source of publicly available summary statistics are indicated in Supplementary Data 1 and the Data Availability Section. No further software were used for data collection.
Data analysis	Software utilized for manuscript<br>(1) GWAS Summary statistics quality control: SumstatsQC v0.1 (https://github.com/maxzylam/SumstatsQC). (2) Global Genetic Correlations: GenomicSEM version 0.0.2 (https://github.com/GenomicSEM/GenomicSEM); (3) K-Medoid clustering and Principal Components Analysis: FactoMineR and FactoExtra R packages (version 1.07.999, Le et al., 2008); fpc R package (version 2.2-9). (4) GWAS-by-Subtraction: GenomicSEM version 0.0.2 (https://github.com/GenomicSEM/GenomicSEM). (5) Local Genetic Correlations: ρ-HESS version 0.5.4 (https://huwenboshi.github.io/hess/local_rhog/); Wrapper script for p-HESS (https://github.com/maxzylam/rho-HESS-wrapper). (6) UMAP/Density Based Scan: uwot package (version 0.1.10); dbscan package (version 1.1.5) (7) Transcriptome Wide Analysis: MAGMA Gene-Based Genome Wide Analysis; MAGMA v1.08 (https://ctg.cncr.nl/software/magma); PoPs Gene Polygenic Priority Score - PoPs v0.1 (https://github.com/FinucaneLab/pops); Summary Statistics Mendelian Randomization / HEIDI -SMR/HEIDI version 1.03 (https://cnsgenomics.com/software/smr/#Download); Summary statistics PrediXcan TWAS - SPrediXcan (Oct 16, 2020 version) (https://github.com/hakyimlab/MetaXcan); FOCUS transcriptomic finemapping - FOCUS (Aug 21, 2020 version) (https://github.com/bogdanlab/focus). (7) Gene Set Analysis: FUMA::GENE2FUNC - FUMA v1.36a https://fuma.ctglab.nl/; WebGestalt - Version 2019 http://www.webgestalt.org/; Gene-Set Enrichment - GSEA 4.10 (https://www.gsea-msigdb.org/gsea/index.jsp); (8) Spatial Temporal Gene Expression: BrainSpan - RNA-Seq Gencode v10 summarized to genes database https://www.brainspan.org/static/download.html; Linear Mixed Model analysis for BrainSpan Data - lmerTest package (version 3.1.3) (9) General biostatistics/data wrangling: R-statistics version 3.6.3 (10) Allen Human Brain Atlas visualizations: BrainScope https://brainscope.lumc.nl/brainscope.

For manuscripts utilizing custom algorithms or software that are central to the research but not yet described in published literature, software must be made available to editors and reviewers. We strongly encourage code deposition in a community repository (e.g. GitHub). See the Nature Portfolio guidelines for submitting code & software for further information.

Data

Policy information about availability of data

All manuscripts must include a data availability statement. This statement should provide the following information, where applicable:

- Accession codes, unique identifiers, or web links for publicly available datasets
- A description of any restrictions on data availability
- For clinical datasets or third party data, please ensure that the statement adheres to our policy

Data Availability:

(1) The GWAS summary statistics for Cognitive Task Performance and Non-Cognitive Factor data generated in this study are available at https://storage.googleapis.com/broad_institute_mlam/brainstorm-v2-local-gencor-1/03_quality_control_sumstatsqc/07_Data_Release_GWAS_Catalog_01/Lam_et_al_2021_CognitiveTaskPerformance.tsv.gz; https://storage.googleapis.com/broad_institute_mlam/brainstorm-v2-local-gencor-1/03_quality_control_sumstatsqc/07_Data_Release_GWAS_Catalog_01/Lam_et_al_2022_NonCognitiveFactor.tsv.gz; The individual genotype data are protected and are not available due to data privacy laws. The processed individual genotype data can be obtained by contracting respective laboratories that contributed to the data. The metadata for Cognitive Task Performance and Non-Cognitive Factor GWAS summary statistics generated in this study are provided in Supplementary Data 1. (2) Previously unpublished GWAS data that was closed access to Biogen Inc. would now be made available. GWAS summary statistics for Education Attainment, General Cognitive Ability, Numeric Reasoning, Pairs Matching, Reaction Time, Verbal Reasoning, and Social Deprivation used in this study are available at https://storage.googleapis.com/broad_institute_mlam/brainstorm-v2-local-gencor-1/03_quality_control_sumstatsqc/07_Data_Release_GWAS_Catalog_01/Biogen_2022_Education_Attainment.tsv.gz; https://storage.googleapis.com/broad_institute_mlam/brainstorm-v2-local-gencor-1/03_quality_control_sumstatsqc/07_Data_Release_GWAS_Catalog_01/Biogen_2022_General_Cognitive_Ability.tsv.gz; https://storage.googleapis.com/broad_institute_mlam/brainstorm-v2-local-gencor-1/03_quality_control_sumstatsqc/07_Data_Release_GWAS_Catalog_01/Biogen_2022_Numeric_Reasoning.tsv.gz; https://storage.googleapis.com/broad_institute_mlam/brainstorm-v2-local-gencor-1/03_quality_control_sumstatsqc/07_Data_Release_GWAS_Catalog_01/Biogen_2022_Pairs_Matching.tsv.gz; https://storage.googleapis.com/broad_institute_mlam/brainstorm-v2-local-gencor-1/03_quality_control_sumstatsqc/07_Data_Release_GWAS_Catalog_01/Biogen_2022_Reaction_Time.tsv.gz; https://storage.googleapis.com/broad_institute_mlam/brainstorm-v2-local-gencor-1/03_quality_control_sumstatsqc/07_Data_Release_GWAS_Catalog_01/Biogen_2022_Social_Deprivation.tsv.gz; https://storage.googleapis.com/broad_institute_mlam/brainstorm-v2-local-gencor-1/03_quality_control_sumstatsqc/07_Data_Release_GWAS_Catalog_01/Biogen_2022_Verbal_Reasoning.tsv.gz. The individual genotype data are available under restricted access, access can be obtained by application to the UK Biobank. The meta-data for the cognitive summary statistics are provided Supplementary Data 1. (3) Polygenic Priority Score gene features used in this study are available at https://github.com/FinucaneLab/pops. Results from the Polygenic Priority Score analysis is available in Supplementary Data 10 (4) eQTL annotations from the Brain e-META, PsychENCODE PEER methodology and PsychENCODE HCP methodology are available at the Summary Statistics Mendelian Randomization website found here (https://yanglab.westlake.edu.cn/software/smr/#DataResource). The Summary statistics mendelian randomization data generated in this study are provided in the Supplementary Data 11a-c. (5) eQTL annotations used in the Transcriptome Wide Analysis as part of the current study is available at https://github.com/hakyimlab/MetaXcan. Results of the TWAS data generated in this study are provided in the Supplementary Data 12a-m. (6) eQTL annotations for FOCUS transcriptome wide fine-mapping analysis used in the study is available at https://github.com/bogdanlab/focus. The FOCUS fine-mapping data generated in this study are provided in the Supplementary Data 13a-b. (7) Gene Ontologies within the Molecular Signature Database 7.2 used in this study are available at https://www.gsea-msigdb.org/gsea/msigdb. Gene set analysis results based on the Molecular Signature Database are provided in the Supplementary Data 16a. (9) DrugBank annotations for WebGestalt analysis is available at. https://go.drugbank.com. Webgestalt analysis results based on the Drugbank annotations are provided in Supplementary Data 16b. (10) Brainspan data used in this study are available at https://www.brainspan.org/static/download.html. The results generated in this study based on the BrainSpan data Supplementary Data 17. (11) Data from the Allen Brain atlas was visualized through the BrainScope Visualizer. Image annotations reported in the current study is available at https://brainscope.lumc.nl/brainscope. Results based on Brainscope visualizer is reported in Supplementary Figures 10-14. (12) GWAS catalog annotations for gene annotations are available at https://www.ebi.ac.uk/gwas/docs/file-downloads. Gene annotations based on GWAS catalog is reported in Supplementary Data 18. (13) All other GWAS summary statistics pertaining to psychopathology traits described in Supplementary Data 1 is available for download at https://pgc.unc.edu/for-researchers/download-results/. Individual level genotype data are restricted due to data privacy laws. Requests for de-identified genotype data should be made to respective data-access committees.

Field-specific reporting

Please select the one below that is the best fit for your research. If you are not sure, read the appropriate sections before making your selection.

☒ Life sciences ☐ Behavioural & social sciences ☐ Ecological, evolutionary & environmental sciences

For a reference copy of the document with all sections, see nature.com/documents/nr-reporting-summary-flat.pdf

Life sciences study design

All studies must disclose on these points even when the disclosure is negative.

Sample size	Sample sizes were based on the original genome-wide association studies with which the GWAS summary statistics were obtained. Original sample sizes are indicated in Supplementary Data 1.
Data exclusions	No data exclusions were made.
Replication	GWAS summary statistics for the largest GWAS for general cognitive ability, and education attainment were used as the input data for the current report. There are no other known data sets in the literature that matches that scale for replication analysis. The crucial results reported at those of the meta-loci. Using multiple simulation approaches we could demonstrate that the main results of the manuscript are likely to be random. As the current study is the first of its kind, direct replication data is not currently available. Nonetheless, we carried out

	additional investigation into the primary results, and found converging evidence from independent eQTL and gene annotation databases that supported the primary findings.
Randomization	No randomization was used to select GWAS summary statistics for cognitive function and psychopathology traits. The study design relies on large-scale GWAS summary statistics in an attempt to uncover biological mechanisms that underlie psychopathology. As this is not a clinical trial nor experimental study, there was no randomization carried out as part of the study design. Moreover, due to the large-scale samples involved, the genetic architecture in itself, represents a form of natural randomization for the current report.
Blinding	No blinding procedures were used for selection of GWAS summary statistics. Blinding procedures typically utilized in clinical trials, are not applicable to a naturalistic study of the genetic architecture of Cognitive Task Performance and Non-Cognitive Factor.

Reporting for specific materials, systems and methods

We require information from authors about some types of materials, experimental systems and methods used in many studies. Here, indicate whether each material, system or method listed is relevant to your study. If you are not sure if a list item applies to your research, read the appropriate section before selecting a response.

Materials & experimental systems

n/a	Involved in the study
☒	☐ Antibodies
☒	☐ Eukaryotic cell lines
☒	☐ Palaeontology and archaeology
☒	☐ Animals and other organisms
☐	☒ Human research participants
☒	☐ Clinical data
☒	☐ Dual use research of concern

Methods

n/a	Involved in the study
☒	☐ ChIP-seq
☒	☐ Flow cytometry
☒	☐ MRI-based neuroimaging

Human research participants

Policy information about studies involving human research participants

Population characteristics	GWAS summary statistics were used in the current report. Sample characteristics of each research sample are available in the original study that the GWAS summary statistics were obtained. PMID codes of the original studies are provided in Supplementary Data 1.
Recruitment	Not Applicable.
Ethics oversight	GWAS summary statistics utilized in the current study are publicly available except for the UK Biobank GWAS summary statistics for cognitive phenotypes and the Townsend Index for Social Deprivation which was approved for use under UK Biobank Approved Application ID 26041 (“Large-Scale Sequencing in the UK Biobank to Facilitate Gene Discovery, Genome Sciences, and Precision Medicine”).

Note that full information on the approval of the study protocol must also be provided in the manuscript.
